# NOR1 loss relates to inflammation and biological ageing in multiple sclerosis motor cortex

**DOI:** 10.1093/braincomms/fcag315

**Published:** 2026-08-14

**Authors:** Buse Unlu Kobya, Aimee Avery, Gabriele DeLuca, Jonathan Pansieri

**Affiliations:** Nuffield Department of Clinical Neurosciences, University of Oxford, Oxford, Oxfordshire, United Kingdom; Nuffield Department of Clinical Neurosciences, University of Oxford, Oxford, Oxfordshire, United Kingdom; Nuffield Department of Clinical Neurosciences, University of Oxford, Oxford, Oxfordshire, United Kingdom; Nuffield Department of Clinical Neurosciences, University of Oxford, Oxford, Oxfordshire, United Kingdom

**Keywords:** multiple sclerosis, ageing, inflammation, NR4A3

## Abstract

Cortical neurodegeneration and glial activation are major drivers of disability progression in multiple sclerosis, but the molecular mechanisms underlying these processes remain poorly defined. Members of the NR4A nuclear receptor family are stress-responsive transcription factors that regulate neuronal survival, inflammatory signalling and cellular adaptation to metabolic and oxidative stress. In particular, neuron-derived orphan receptor 1 (NR4A3) has been implicated in neuroprotective and stress-adaptive responses in other neurological conditions, but its role in multiple sclerosis has not been investigated. Given its dual involvement in neuronal stress regulation and inflammatory modulation, neuron-derived orphan receptor 1 represents a plausible molecular link between chronic neuroinflammation, biological ageing and cortical neurodegeneration in multiple sclerosis. Here, we examined neuron-derived orphan receptor 1 expression in the motor cortex of post-mortem multiple sclerosis (*n* = 50) and control (*n* = 10) cases across lesional and non-lesional cortical layers of the motor cortex. Neuron-derived orphan receptor 1 was expressed by various cell types but predominantly localized to the neuronal cytoplasm and was reduced in multiple sclerosis non-lesional grey matter compared with controls, in particular in the functionally relevant layer V. In layer V, neuron-derived orphan receptor 1 loss was linked to exacerbated astrocytic (GFAP+) and microglial/macrophage (CD68+) expression, and higher levels of senescence markers (p19^INK4d, p21^CIP1), suggesting that loss of neuron-derived orphan receptor 1 relates to neuroinflammation and biological ageing processes. In contrast, in layer III, neuron-derived orphan receptor 1 expression was positively associated with neuronal density (NeuN) and Nurr1 expression, a transcription factor from the same NR4A family with established neuroprotective functions. Together, our findings identify neuron-derived orphan receptor 1 as a previously unrecognized, layer-specific regulator of cortical pathology in multiple sclerosis. Loss of neuron-derived orphan receptor 1 in deep-layer projection neurons aligns with inflammatory amplification and ageing-associated stress responses, whereas its preservation in upper layers may support neuronal integrity and neuroprotective signalling. These data position neuron-derived orphan receptor 1 as a candidate molecular node linking neuroinflammation, senescence and cortical neurodegeneration in progressive multiple sclerosis, and support that strategies aimed at preserving or restoring neuron-derived orphan receptor 1 activity may support neuron resilience and mitigate cortical disease progression in this incurable disease.

## Teaser

Unlu Kobya *et al*. report that neuron-derived orphan receptor 1 is reduced in the motor cortex of multiple sclerosis and is associated with increased inflammation and biological ageing. Loss of this receptor suggests that restoring its function may represent a potential strategy to mitigate neurodegeneration in multiple sclerosis.

## Graphical Abstract

**Graphical Abstract:**
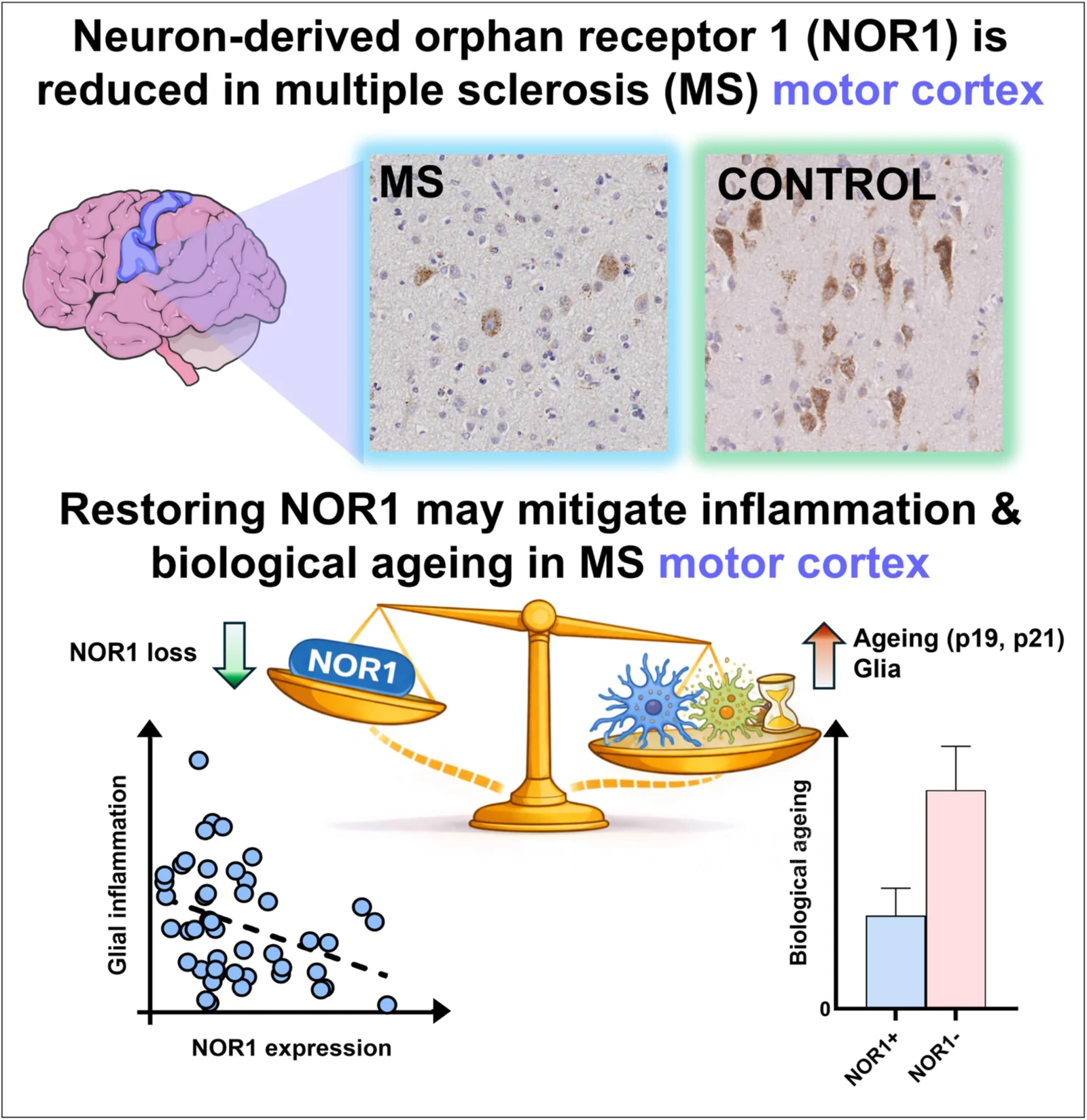

## Introduction

Multiple sclerosis is a chronic inflammatory and neurodegenerative disease of the CNS, characterized by demyelination, glial activation and progressive neurodegeneration. While white matter (WM) demyelinated lesions have traditionally been considered the pathological hallmark of the disease, increasing attention has been given to grey matter (GM) pathology, particularly in the cerebral cortex, as a major contributor to disease progression and irreversible disability in progressive multiple sclerosis phenotypes.^1-3^ Among cortical regions, the motor cortex is particularly vulnerable, with studies consistently reporting neuronal damage, glial activation and synaptic degeneration, even in areas without overt demyelination.^4,5^ These non-lesional areas often harbour biologically significant pathological changes that reflect chronic, diffuse neuroinflammatory and neurodegenerative processes.^6,7^

In recent years, the concept of cortical pathology in multiple sclerosis has expanded to include not only inflammation and demyelination but also metabolic stress, cellular senescence and dysregulation of transcriptional homeostasis.^8-10^ However, the molecular mediators linking these mechanisms to progression remain incompletely defined. Among the candidate regulators of stress and survival pathways in the CNS are members of the NR4A (nuclear receptor subfamily 4A) family of orphan nuclear receptors, namely Nur77 (NR4A1), Nurr1 (NR4A2) and NOR1 (NR4A3). These transcription factors are rapidly induced by cellular stress and neuronal activity and are involved in a wide range of homeostatic functions, including synaptic regulation, inflammatory control and cell survival.^11-13^ In progressive multiple sclerosis, our recent work has implicated Nurr1 in neuroprotective mechanisms, showing association with higher neuronal density and reduced immune response, supporting its role as an endogenous protective factor.^14^ By contrast, NOR1, despite its structural similarity to Nurr1 and overlapping transcriptional networks, remains largely less explored in neurological conditions. However, it was suggested that NOR1 may modulate apoptosis and regeneration following injury,^15^ and recent work shows stress-responsive regulation supporting a potential neuroprotective role.^16^ NOR1 expression was also altered under oxidative or degenerative conditions in non-neural tissues, and its interaction with regulators of cellular senescence has been observed in aged cells.^17^ These lines of evidence support a biological plausibility that NOR1 may contribute to mechanisms of accelerated ageing in the human brain. In this context, cellular senescence markers such as p19^INK4d (termed p19) and p21^CIP1 (termed p21) have emerged as important indicators of ageing-related stress responses and chronic tissue dysfunction in neurological conditions.^18^ Given that neuronal vulnerability, chronic inflammation and features of biological ageing and senescence converge in progressive multiple sclerosis, we hypothesized that NOR1 could function as an integrative transcriptional node at the intersection of these pathological domains.

In this neuropathological study, we quantified NOR1 expression across cortical layers of the motor cortex in post-mortem multiple sclerosis (*n* = 50) and control (*n* = 10) brains, assessing its relationship to astrocytic and microglial expression (GFAP+, CD68+), Nurr1 expression (Nurr1), neuronal density (NeuN) and biological ageing markers (p19, p21). We hypothesized that altered NOR1 expression may reflect a breakdown of neuroprotective mechanisms, contributing to cortical pathology in progressive multiple sclerosis, wherein treatment options are currently limited.

## Materials and methods

### Study population

This study analysed post-mortem human brain tissue from 50 individuals with pathologically confirmed multiple sclerosis and 10 non-neurological controls, obtained from the UK Multiple Sclerosis Tissue Bank (Table 1 and Supplementary Table 1). All samples were collected with informed consent and ethical approval (REC 08/MRE09/31+5) in accordance with the UK Human Tissue Act (2004). Brain weight was recorded prior to tissue sampling. All tissue samples were obtained from the precentral gyrus, corresponding to the primary motor cortex.

**Table 1:** Case selection and demographical features used for NOR1 assessments in the multiple sclerosis and control cohort. Comparisons using Mann–Whitney U-test were used to compare clinical features

	Multiple sclerosis cases (*n* = 50)	Controls (*n* = 10)	*P*-value
**Age at death (years)**	65.6 (range 40–92)	77.7 (range 68–91)	0.017
**Duration of disease (years)**	30.7 (range 8–58)	n/a	n/a
**Sex**	M = 15 F = 35	M = 6 F = 4	0.14
**Clinical course**	PPMS: 8; SPMS: 41; RRMS: 1	n/a	n/a
**Brain weight (g)**	1170 (range 800–1557)	1331 (range 1072–1628)	0.01
**Time to wheelchair (years)**	18.8 (range 2−50)	n/a	n/a
**PM interval (h)**	17.1 (range 6–38)	27.5 (range 10–52)	0.06

Multiple sclerosis Cohort at Extremes of infragranular NOR1 Expression (p19/p21 assessment)			
	Multiple sclerosis: low NOR1 (*n* = 10)	Multiple sclerosis: high NOR1 (*n* = 10)	
**Age at death (years)**	66.9 (range 55–86)	67.2 (range 40–92)	0.67
**Duration of disease (years)**	28.3 (range 8–38)	31.6 (range 13–58)	0.86
**Sex**	M = 5 F = 5	M = 3 F = 7	0.65
**Clinical course**	PPMS:3; SPMS: 7	PPMS: 3; SPMS: 7	n/a
**Brain weight (g)**	1164 (range 1050–1370)	1191 (range 935–1364)	0.43
**Time to wheelchair (years)**	18.0 (range 2–36)	20.3 (range 4–50)	0.67
**PM interval (h)**	19.6 (range 9–38)	18.5 (range 7–27)	0.93

A subset of multiple sclerosis cases representing the extremes of NOR1 expression in deep layers of multiple sclerosis motor cortex (multiple sclerosis; *n* = 10 low; *n* = 10 high) was selected for additional analyses of senescence markers (p19, p21), matching for clinical features.

### Immunohistochemistry and immunofluorescence

Formalin-fixed paraffin-embedded sections (6 µm) of motor cortex were cut on a microtome and mounted on charged slides. After deparaffinization and heat-induced antigen retrieval (see Supplementary Table 2), sections were incubated with primary antibodies targeting NOR1, neurons (NeuN), Aβ (4G8), astrocytes (GFAP), microglia/macrophages (Iba1, CD68), Nurr1 and senescence markers (p19, p21) following standard procedures.

Briefly, detection was achieved by a conventional peroxidase-based system using 3,3′-diaminobenzidine (DAB; Dako REAL EnVision System, #K5007). All sections were counterstained with haematoxylin. Non-specific background was minimized using a commercial serum-free protein block (Dako X0909). Negative controls included omission of primary or secondary antibodies on adjacent sections. Importantly, to confirm the specificity of NOR1 staining, additional and independent control experiments included no primary antibody (data not shown), isotype-matched IgG antibodies and a competitive blocking assay using a recombinant NOR1 control fragment (aa 377–455) directed against the same epitope as the primary antibody (aa 332–626, Supplementary Fig. 1).

Immunofluorescence (IF) was performed on a subset of multiple sclerosis cases (*n* = 2) and controls (*n* = 2), using the same panel of primary antibodies, along with additional markers to identify endothelial cells (CD31/CD34), Aβ (4G8), astrocytes (GFAP) and microglia (TMEM119). Fluorophore-conjugated secondary antibodies (Alexa Fluor 488, 594, 647; Invitrogen) were applied. Lipofuscin and background autofluorescence were quenched using TrueBlack (Biotium #23007).

For dual chromogenic/IF labelling, IF staining was performed first, followed by antibody stripping using a validated antibody removal kit following previously published procedures,^19^ and then re-stained with DAB-based detection. Negative controls confirmed complete antibody removal prior to DAB application (Supplementary Fig. 2).

To ensure robust comparison between markers, quantification was performed on adjacent tissue sections, and corresponding fields of view (FOVs) were accurately aligned using image registration tools. This approach ensured that identical cortical areas were sampled across all markers.

#### Demyelination status

Motor cortical demyelination was assessed using proteolipid protein staining to distinguish demyelinated GM or WM lesions from adjacent non-lesional GM (NLGM) and non-lesional WM (NLWM). Only total demyelination was considered as demyelinated areas. For GM lesions, FOVs were defined using the same strategy as for NLGM and compared with corresponding NLGM cortical layers, with NLGM FOVs placed at least 600 µm from the lesion edge. For WM lesions, five FOVs per lesion were arranged around the centre of the lesion and compared with NLWM FOVs placed at least 600 µm from the lesion edge. According to well-established classification criteria^20^ (Supplementary Fig. 3), demyelinated lesions were classified into leucocortical (extending from the subcortical WM into the adjacent cortex), subpial (cortical demyelination spreading inward from the pia mater layer I to layer III), pancortical (involving the full thickness of the cortex without entering the underlying WM), intracortical (demyelination in GM layers without spreading to pia or WM) and WM lesions (confined to the WM without cortical involvement).

#### Quantification of NOR1 expression

Digital whole-slide images were acquired using the Aperio ScanScope AT Turbo scanner at ×400 magnification. Spatial quantification of NOR1 expression was conducted using a previously established approach: predefined cortical trajectories were drawn perpendicular to the pial surface, across which FOVs were consistently placed within NLGM layers and, when present, adjacent WM. Layer III contained four FOVs per trajectory, while other layers had two. NLGM layers I–III were classified as supragranular and IV–VI as infragranular. A total of over 12 000 FOVs were analysed.

NOR1 expression within each FOV was quantified using QuPath software by extracting chromogen-positive pixels through a supervised pixel classification approach. Specifically, a classifier was trained on 24 manually annotated regions (500 × 500 µm) from randomly selected multiple sclerosis and control cases, in which pixels were manually labelled as either NOR1 positive or background. Classification was performed using a Random Trees algorithm with multiscale feature extraction at full resolution (0.25 µm/pixel). This approach enabled robust and consistent detection of chromogen-positive pixels across all samples, expressed as area coverage (%).

#### Quantification of NOR1+ neurons

Neurons with cytoplasmic or nuclear NOR1 expression were manually counted in the same FOVs as described above in the NLGM layer III and V (Supplementary Fig. 4). For cytoplasmic counts, only neurons that met morphological criteria were included^14,21^: pyramidal shape, soma size ≥100 µm^2^ and a clearly defined nucleus and nucleolus. For nuclear counts, only neurons that met strict morphological criteria were included: pyramidal shape, soma size ≥100 µm^2^, accompanied by strong, round-shaped intensity of the nuclear staining. Over 9000 neurons and 2000 neurons were manually counted for neurons with cytoplasmic and nuclear NOR1 expression, respectively, with results expressed as cells/mm^2^. Importantly, classification as NOR1+ neurons was based on the presence of a clear and unequivocal chromogenic signal, as defined by the pixel classification approach described above. Cells with faint or ambiguous staining were excluded from the analysis.

#### Quantification of plaque-associated NOR1+ neuritic profiles

Plaque-associated neuritic profiles showing NOR1 expression (termed NOR1^+^ neuritic profiles) were quantified in the same FOVs used for other NOR1 expression analyses. NOR1^+^ neuritic profiles were defined as heterogeneous and numerous round or ovoid structures with compact morphology, identifiable by chromogenic signal intensity and shape. Only neuritic profiles with a diameter between 35 and 100 μm were included in the analysis. Smaller aggregates (<25 μm) or irregularly shaped deposits were excluded to avoid including diffuse or non-specific staining. The number of neuritic profiles per mm^2^ was calculated based on the total area of the analysed FOVs, and values were averaged across multiple fields per case.

#### Quantification of brain cell markers, Nurr1 and biological ageing markers

Neuronal density was quantified by automated counting of NeuN+ cells per FOV in layers III and V using QuPath software. Cell detection was performed using the ‘Positive Cell Detection’ algorithm with standardized parameters across all samples, based on optical density (DAB) thresholds to identify neuronal cells. Key parameters included a requested pixel size of 3 µm, nucleus area thresholds of 50–600 µm^2^ and intensity classification based on DAB optical density. Additional parameters included separation of touching cells based on shape. This automated method was validated against manual counts in a subset of cases, yielding similar values (Supplementary Fig. 5).

Astrocytes (GFAP+), microglia/macrophages (CD68+), Nurr1 and biological ageing p21^CIP1 (CDKN1A/p21^CIP1) expression were quantified in adjacent sections using similar methods as for NOR1 expression, expressed as area coverage (%).

Concerning p19^INK4a, given its known role in regulating nuclear senescence pathways and its predominant nuclear localization, positive nuclei were manually counted across the same FOVs used for other markers and expressed as positive cells/mm^2^.

#### Image alignment for DAB/IF co-localization

In sections stained sequentially for DAB and IF, high-resolution images were aligned using a two-step process. Fluorescent scans were initially co-registered with the DAB brightfield images using QuPath to define matching landmarks. Warping was then refined in ImageJ using the Warpy plugin, which performs non-linear alignment based on tissue landmarks to account for minor deformations introduced during antibody stripping and reprocessing, as previously published.^22^ This allowed precise overlay of cell-specific markers and transcription factor expression.

### Statistical analyses

Clinical features comparing multiple sclerosis and control groups were assessed using the non-parametric Mann–Whitney U-test.

A generalized linear model was conducted to evaluate the impact of disease status (disease versus control) on NOR1 expression in NLWM and NLGM, including post-mortem interval (PMI) and age-at-death as covariates. Despite the inclusion of these covariates, neither PMI nor age-at-death significantly influenced the NOR1 results. The NOR1 dataset was log-transformed to address non-normality prior to conducting a stepwise regression analysis with age, PMI and sex as predictors. The stepwise regression analysis indicated that none of these predictors significantly impacted NOR1 expression, leading to their exclusion from further analysis.

To compare NOR1 scores and neuronal density, glial and Nurr1 expression between multiple sclerosis and control groups, the Mann–Whitney U-test was employed, where the Bonferroni–Dunn correction was applied to adjust for Type I errors when relevant.

Differences between lesional and corresponding non-lesional areas within each case were assessed using the two-tailed Wilcoxon matched-pairs signed-rank test.

Correlation analyses to evaluate relationships between NOR1 expression and clinical features and other markers were performed using Spearman rank correlation coefficients.

Data are presented with ± standard error of the mean (SEM). Two-tailed tests were applied where appropriate, with *P*-values < 0.05 considered statistically significant. Statistical analyses were conducted using GraphPad Prism (version 9.5.1) and SPSS (version 29).

## Results

### Demographic features of the cohort

Demographic and clinical features of the study cohort are presented in Table 1. The multiple sclerosis group included 50 cases (35 female, 15 male), while the control group included 10 cases (4 female, 6 male). Most of the multiple sclerosis cases had a secondary progressive course (*n* = 41), with eight primary progressive multiple sclerosis and one relapsing-remitting multiple sclerosis case. Time from onset to wheelchair use was available for a subset of cases, with a mean of 18.8 ± 11.2 years.

Control cases were older than multiple sclerosis cases (age-at-death; multiple sclerosis: 63.9 ± 12.0 years old, control: 77.7 ± 6.6 years old; *P* = 0.002), while brain weight was reduced in multiple sclerosis compared with controls (brain weight; multiple sclerosis: 1153 ± 141 g, control: 1331 ± 184 g; *P* = 0.01). PMI and sex did not differ between multiple sclerosis and controls (PMI: *P* = 0.06; sex: *P* = 0.14).

Demyelinated lesions were detected in 60% of multiple sclerosis cases (30 out of 50). Subpial lesions were found in 52%, pancortical lesions in 6%, leucocortical lesions in 4%, intracortical lesions in 2% and WM lesions in 6% of multiple sclerosis cases. No demyelinated lesions were found in controls.

### Cellular and subcellular distribution of NOR1 in multiple sclerosis and control motor cortex

**Figure 1:**
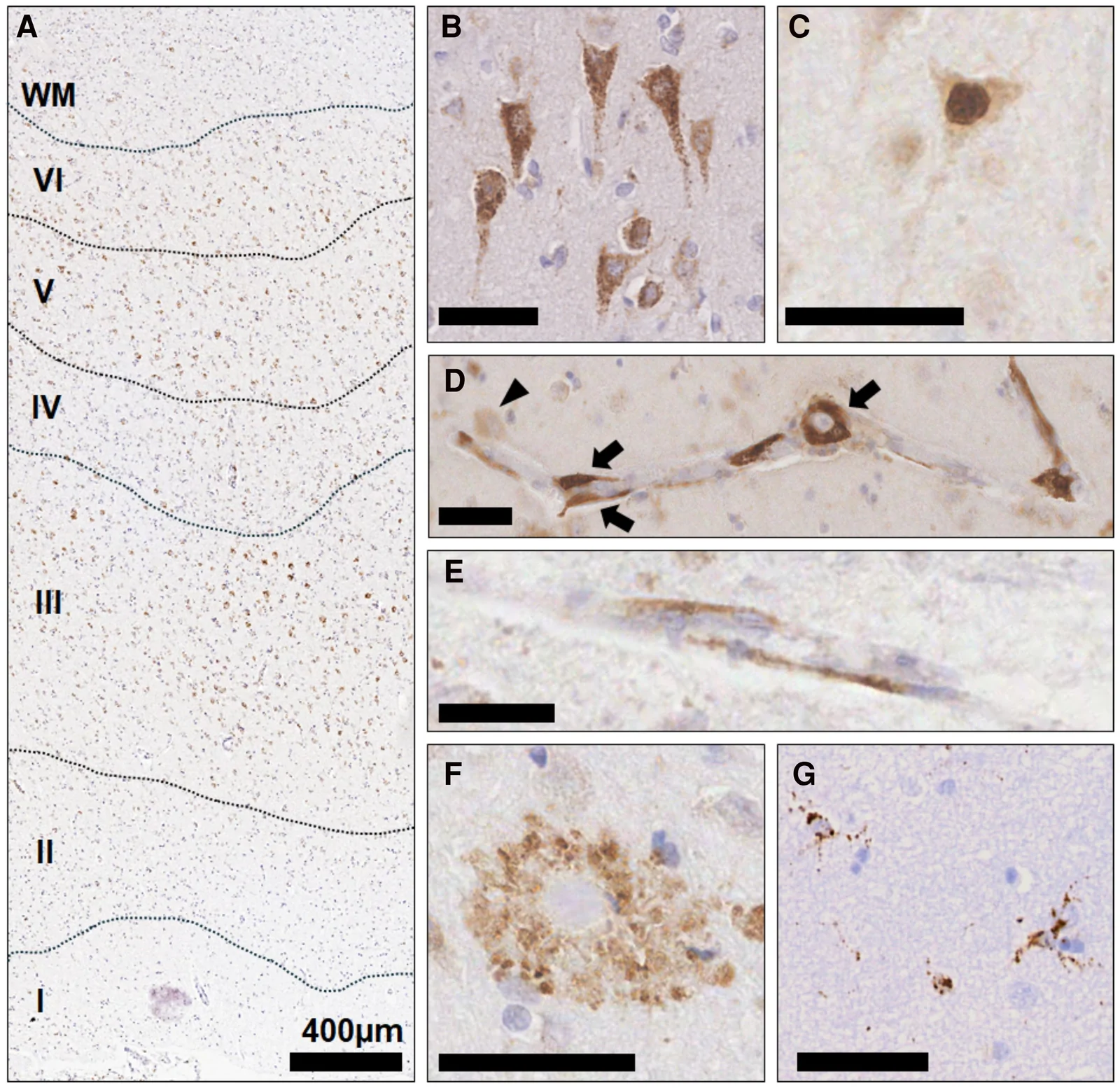
**NOR1 is expressed in various brain cells in multiple sclerosis and control motor cortex.** (**A**) Representative NOR1 expression in multiple sclerosis motor cortex with specific layers from I to VI of the non-lesional grey matter. (**B and C**) Representative pictures of the predominant neuronal NOR1 pattern in layer V pyramidal neurons, showing (**B**) a cytoplasmic and (**C**) a nuclear pattern. (**D and E**) Representative pictures of vascular-related NOR1 expression, suggesting (**D**) pericyte expression (elongated, flattened and fusiform cells along vessel walls, arrows) with adjacent neuron (arrowhead) and (**E**) endothelial (inner wall capillary, flattened morphology) expression. (**F**) Representative picture of a plaque-associated NOR1+ neuritic profile. (**G**) Representative picture of NOR1+ glial expression (compact cell body with thin, ramified processes). (NOR1 = neuron-derived orphan receptor 1; scale bars 50 µm in **B–G**; brown colour represents NOR1-positive staining, purple colour represents haematoxylin counterstaining).

Qualitative assessment revealed a heterogeneous spatial and cellular distribution of NOR1 in the GM of the motor cortex of both multiple sclerosis and control cases (Fig. 1 and Supplementary Figs 6–9).

NOR1 was predominantly expressed in pyramidal neurons within cortical layers III and V, which exhibited robust cytoplasmic staining (Fig. 1A and B and Supplementary Figs 6 and 7) accompanied by nuclear localization in layer V–VI (Fig. 1C).

NOR1 immunoreactivity was also observed in vascular-associated mural cells, including elongated, fusiform pericyte-like cells encasing microvessels (Fig. 1D) and occasionally in flattened endothelial profiles lining the vascular lumen (Fig. 1E). These vascular expression patterns were present across both multiple sclerosis and control groups and did not appear restricted to perivascular inflammatory foci.

Surprisingly, dense NOR1+ deposits resembling neuritic plaques were identified in a subset of multiple sclerosis and control cortices, identified by co-localization with 4G8+ amyloid plaques (Fig. 1F and Supplementary Fig. 7). These structures displayed focal, compact accumulation of NOR1 immunoreactivity within dystrophic processes. Importantly, given the propensity of amyloid plaques to sequester surrounding proteins, competition assays confirmed the specificity of NOR1 staining in these plaques (Supplementary Fig. 2). However, AT8-positive (phospho-tau) neuritic plaques were not detected in our multiple sclerosis cohort, and thus co-localization with tau could not be assessed (data not shown). Consequently, the precise localization of NOR1 within these structures (intraneuritic versus extracellular) cannot be definitively established, which we defined as plaque-associated NOR1+ neuritic profiles.

NOR1 expression was occasionally observed in non-neuronal cells displaying morphologies consistent with microglia, characterized by small somata with ramified processes and punctate staining patterns (Fig. 1G) reminiscent of microglial profiles^23^ and validated by co-labelling with TMEM119+ microglial cells (Supplementary Fig. 8). In addition, NOR1 signal was noted in association with GFAP+ astrocytes, suggestive of astrocytic localization (Supplementary Fig. 9).

Of note, in the WM, NOR1 expression was generally low but detectable in a subset of cases, where it appeared in small, irregularly shaped cells consistent with glial morphology. The staining pattern suggests possible expression by microglia/macrophages and/or oligodendrocyte lineage cells. Demyelinated lesions exhibited similar NOR1 patterns to those observed in adjacent non-lesional areas.

### NOR1 expression is reduced in multiple sclerosis deep cortical layers

**Figure 2:**
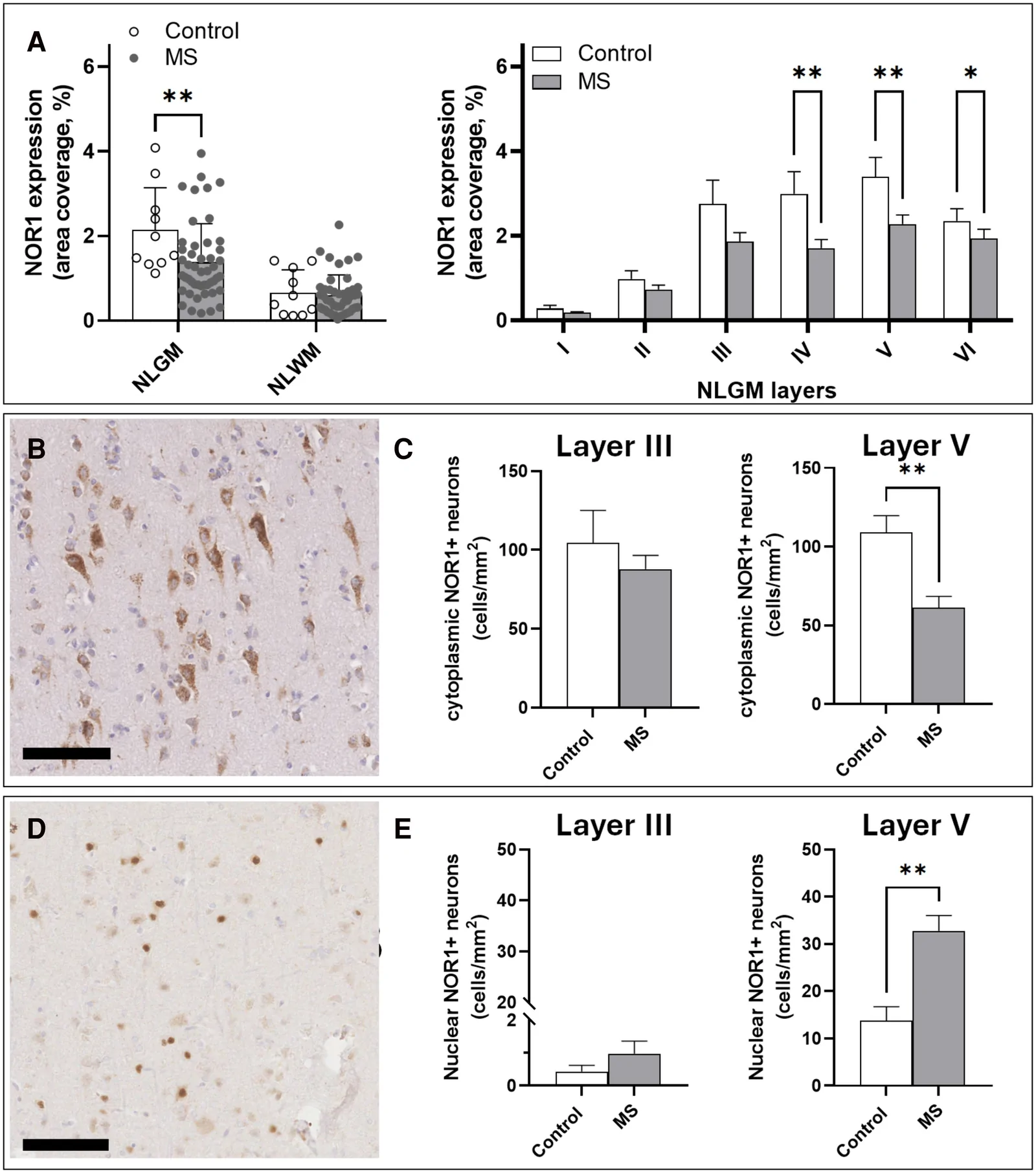
**NOR1 is reduced in infragranular layers of the multiple sclerosis motor cortex.** (**A**) NOR1 expression was reduced in non-lesional grey matter but not in the non-lesional white matter of the multiple sclerosis motor cortex (*n* = 50) compared with control cases (*n* = 10), in particular in deep cortical layers IV to VI. (**B**) Representative picture of NOR1 expression in the cytoplasm of layer V neurons. (**C**) Cytoplasmic NOR1+ neuronal density is reduced in layer V but not in layer III of multiple sclerosis motor cortex versus controls. (**D**) Representative picture of NOR1 expression in the nucleus of layer V neurons. (**E**) Nuclear NOR1+ neuronal density is increased in layer V of multiple sclerosis motor cortex versus controls and is rarely expressed in layer III of both multiple sclerosis and controls. (Mann–Whitney U-test with Bonferroni correction; results are presented as mean ± SEM; *P < 0.05; **P < 0.01; NLGM = non-lesional grey matter; NLWM = non-lesional white matter; NOR1 = neuron-derived orphan receptor 1; scale bar 100 µm; in (**B, C and D**) brown colour represents NOR1-positive staining, purple colour represents haematoxylin counterstaining).

The extent of total NOR1 expression in NLGM and NLWM was quantified in multiple sclerosis cases and controls (Fig. 2).

Compared with controls, NOR1 expression was reduced in NLGM but not the NLWM of the multiple sclerosis motor cortex (Fig. 2A; area coverage in %; NLWM, multiple sclerosis: 0.67, CI95% 0.47–0.86 versus controls: 0.66, CI95% 0.27–1.04; *P* = 0.87; NLGM, multiple sclerosis: 1.36, CI95% 1.16–1.78 versus controls: 2.15, CI95% 1.43–2.85; *P* = 0.008). Layer-specific quantification revealed that NOR1 loss was more pronounced in infragranular layers IV–VI (Fig. 2A; multiple sclerosis versus controls: layer I, *P* = 0.09; layer II, *P* = 0.07; layer III, *P* = 0.10; layer IV, *P* = 0.003; layer V, *P* = 0.007; layer VI, *P* = 0.047).

No relationship was found between total NOR1 expression and PMI, brain weight, age at death, disease duration, sex or time from onset to wheelchair in multiple sclerosis or controls, where applicable (Supplementary Fig. 10). In multiple sclerosis cases, no difference in total NOR1 expression was found between non-lesional and lesional areas, in both GM and WM (Supplementary Fig. 11).

Quantitative measures of NOR1+ neurons were then performed focusing on layer III and layer V, given the preferential location of NOR1 in pyramidal neurons in these layers (Fig. 2B–E).

Comparing the density of NOR1+ neurons according to their cytoplasmic localization revealed a reduction of cytoplasmic NOR1+ neurons in multiple sclerosis compared with controls only in layer V (Fig. 2C; cell density in cells/mm^2^; layer III, multiple sclerosis: 87.8, CI95% 70.3–105.4 versus controls: 104.4, CI95% 51.1–157.7; *P* = 0.47; layer V, multiple sclerosis: 61.2, CI95% 46.6–75.5 versus controls: 108.9, CI95% 81.2–136.7; *P* = 0.005). Conversely, comparing the density of NOR1+ neurons according to their nuclear localization revealed an increase in nuclear NOR1+ neurons in multiple sclerosis compared with controls only in layer V, while in layer III, nuclear NOR1+ neurons were almost absent in both multiple sclerosis and controls (Fig. 2D and E; cell density in cells/mm^2^; layer III, multiple sclerosis: 0.97, CI95% 0.2–1.7 versus controls: 0.43, CI95% 0.0–0.86; *P* = 0.92; layer V, multiple sclerosis: 32.8, CI95% 26.3–39.3 versus controls: 13.7, CI95% 6.9–20.5; *P* = 0.0098). Interestingly, cytoplasmic NOR1+ neuronal density was negatively correlated with nuclear NOR1+ neuronal density (data not shown, *r* = −0.49, *P* = 0.0005), supporting a reciprocal pattern consistent with the main findings.

No relationship was found between nuclear or cytoplasmic NOR1+ neuronal density and PMI, brain weight, age at death, disease duration, sex or time from onset to wheelchair in multiple sclerosis or controls, where applicable. To exclude a simple density artefact, we repeated the analysis using the ratio of NOR1+ neurons to the total number of neurons rather than counts, wherein our findings were similar (data not shown). Of note, exclusion of the single relapsing-remitting multiple sclerosis case did not alter the differences in NOR1 expression observed between multiple sclerosis and control cases.

### NOR1 relates to neuronal density and neuroprotective Nurr1 in multiple sclerosis superficial cortical layers

To explore the relationship between NOR1 expression and neurodegeneration, we analysed neuronal density and Nurr1 expression in cortical layers III and V of multiple sclerosis and control cases, as Nurr1 is a related NR4A receptor with potential neuroprotective functions^14^ that may interact with NOR1 in neuronal survival.

**Figure 3:**
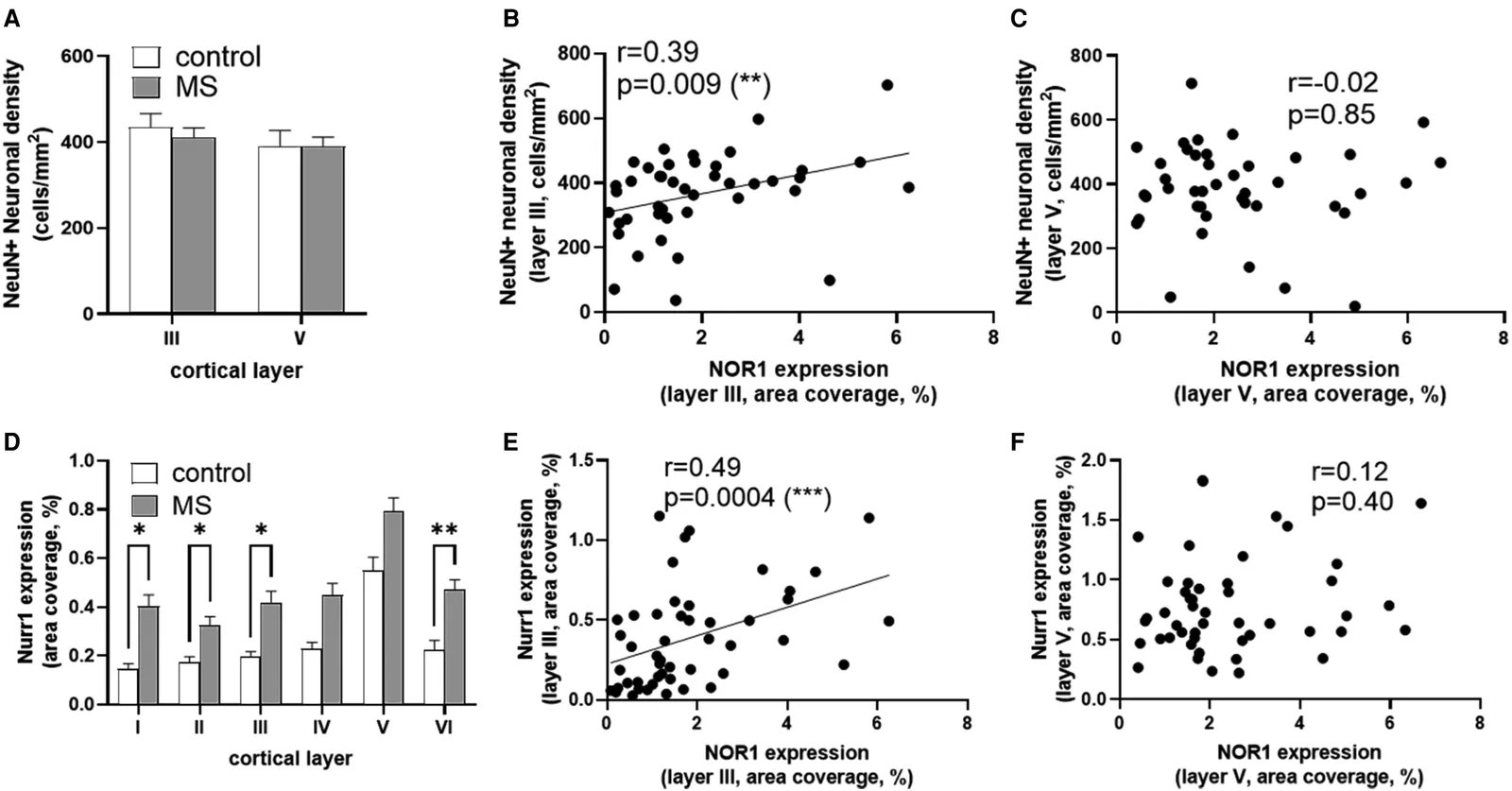
**NOR1 preservation in layer III of multiple sclerosis motor cortex relates to neuronal density and Nurr1 expression.** (**A**) Quantification of neuronal density in cortical layers III and V in multiple sclerosis (*n* = 50) and control cases (*n* = 10) revealed no differences between groups. (**B**) Within the multiple sclerosis cohort, NOR1 expression in layer III was associated with neuronal density, whereas (**C**) no relationship was found in layer V. (**D**) Nurr1 expression was elevated in multiple sclerosis compared with controls, consistent with previous findings and (**E**) NOR1 expression was associated with Nurr1 in layer III but (**F**) not in layer V. (Mann–Whitney U-test with Bonferroni correction in **A and D**; Spearman correlation in **B, C**, **E and F**; histograms are presented as mean ± SEM; *P < 0.05; **P < 0.01; ***P < 0.001; NOR1 = neuron-derived orphan receptor 1; NeuN = neuronal nuclear antigen in **(B, C, E and F)**, each data point represents the values for a single multiple sclerosis case).

Overall neuronal density was similar between multiple sclerosis and control cases, as previously published.^24^ Within the multiple sclerosis cohort, NOR1 expression in layer III was associated with neuronal density (*r* = 0.39, *P* = 0.009) while in layer V, no relationship was found (*r* = 0.02, *P* = 0.85; Fig. 3A–C). However, of note, nuclear NOR1+ neuronal density was associated with neuronal density in layer V (Supplementary Fig. 12).

Nurr1 expression was increased in multiple sclerosis compared with controls, as previously published.^14^ Within the multiple sclerosis cohort, NOR1 expression was positively related to Nurr1 expression in layer III, while in layer V, no relationship was found (Fig. 3D–F).

No additional relationship between nuclear or cytoplasmic NOR1+ neuronal density was found with neuronal density and Nurr1 expression (Supplementary Fig. 12).

### NOR1 loss relates to exacerbated inflammation in multiple sclerosis motor cortex

**Figure 4:**
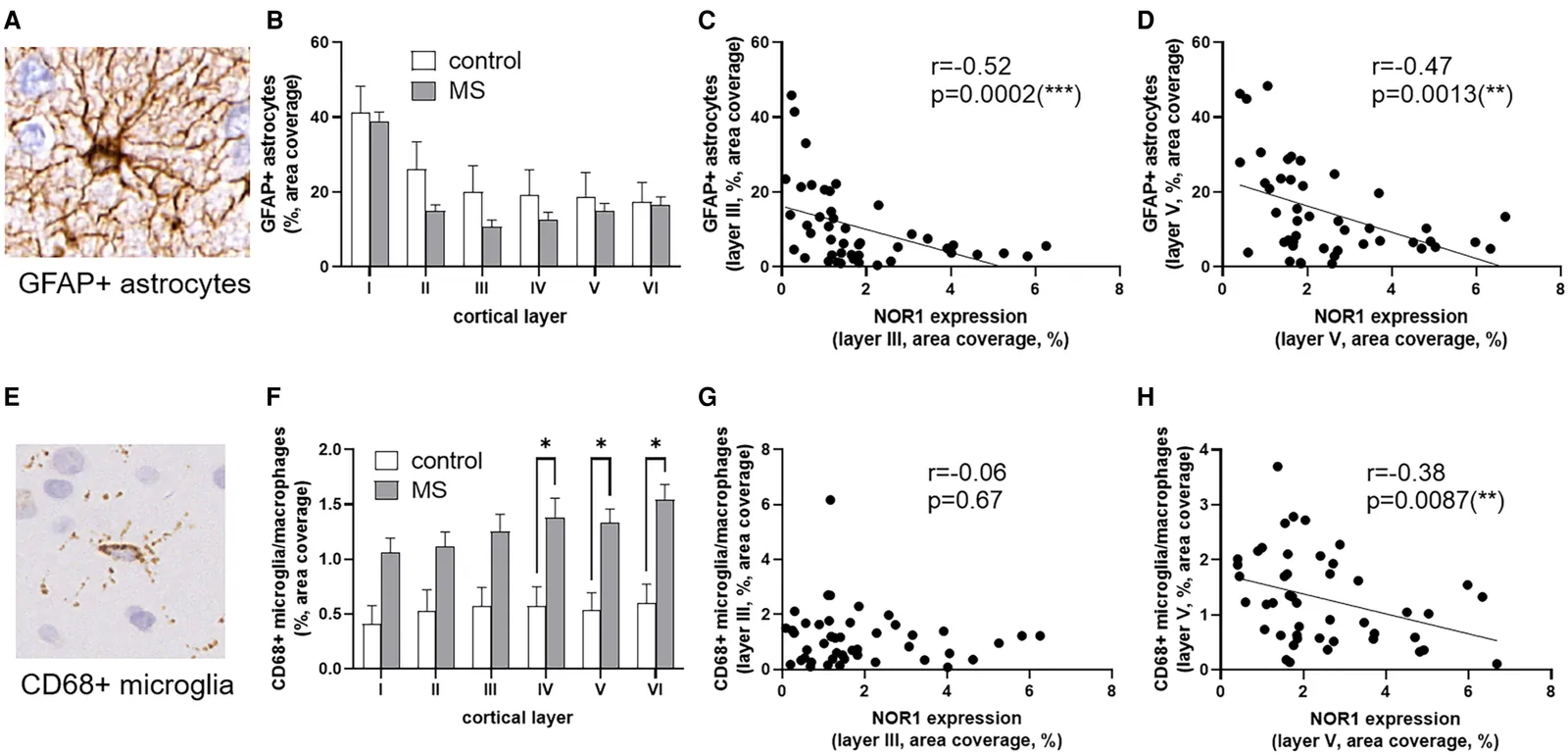
**NOR1 loss is related to increased glial inflammation in layer V of the multiple sclerosis motor cortex.** (**A**) Representative image of a GFAP^+^ astrocytes. (**B**) No difference between multiple sclerosis (*n* = 50) and controls (*n* = 10) in GFAP expression was found. (**C and D**) Within multiple sclerosis cohort, a negative correlation between GFAP and NOR1 expression was found in both layers III and V. (**E**) Representative image of a CD68^+^ microglia. (**F**) CD68 expression was increased in infragranular layers of the multiple sclerosis motor cortex compared with controls. (**G and H**) Within multiple sclerosis cohort, a negative correlation between CD68^+^ expression and NOR1 expression, restricted to layer V. (Mann–Whitney U-test with Bonferroni correction in **B and F;** Spearman correlation in **C, D**, **G and H;** *P < 0.05; **P < 0.01; GFAP = glial fibrillary acidic protein; NOR1 = neuron-derived orphan receptor 1; CD68 = Cluster of Differentiation 68; in (**A and E**) brown colour represents GFAP+ and CD68+ staining, respectively, and purple colour represents haematoxylin counterstaining; in (**C, D, G and H**), each data point represents the values for a single multiple sclerosis case).

To evaluate whether NOR1 expression is related to glial inflammation, we quantified its relationship with markers of reactive astrocytes (GFAP, Fig. 4A–D) and microglia/macrophages (CD68, Fig. 4E–H) in multiple sclerosis motor cortex.

GFAP+ astrocyte burden was not significantly different between multiple sclerosis and control cases (all comparisons, *P* > 0.16). However, in multiple sclerosis, GFAP immunoreactivity was negatively correlated with NOR1 expression in both layer III and layer V.

CD68+ microglia/macrophages were significantly increased in multiple sclerosis cases compared with controls (Fig. 4F; multiple sclerosis versus controls: layer I, *P* = 0.06; layer II, *P* = 0.17; layer III, *P* = 0.08; layer IV, *P* = 0.04; layer V, *P* = 0.03; layer VI, *P* = 0.02), in particular in infragranular layers. Importantly, CD68 expression was negatively correlated with NOR1 expression in layer V.

### Plaque-associated NOR1+ neuritic profiles reflect age-associated mechanism

**Figure 5:**
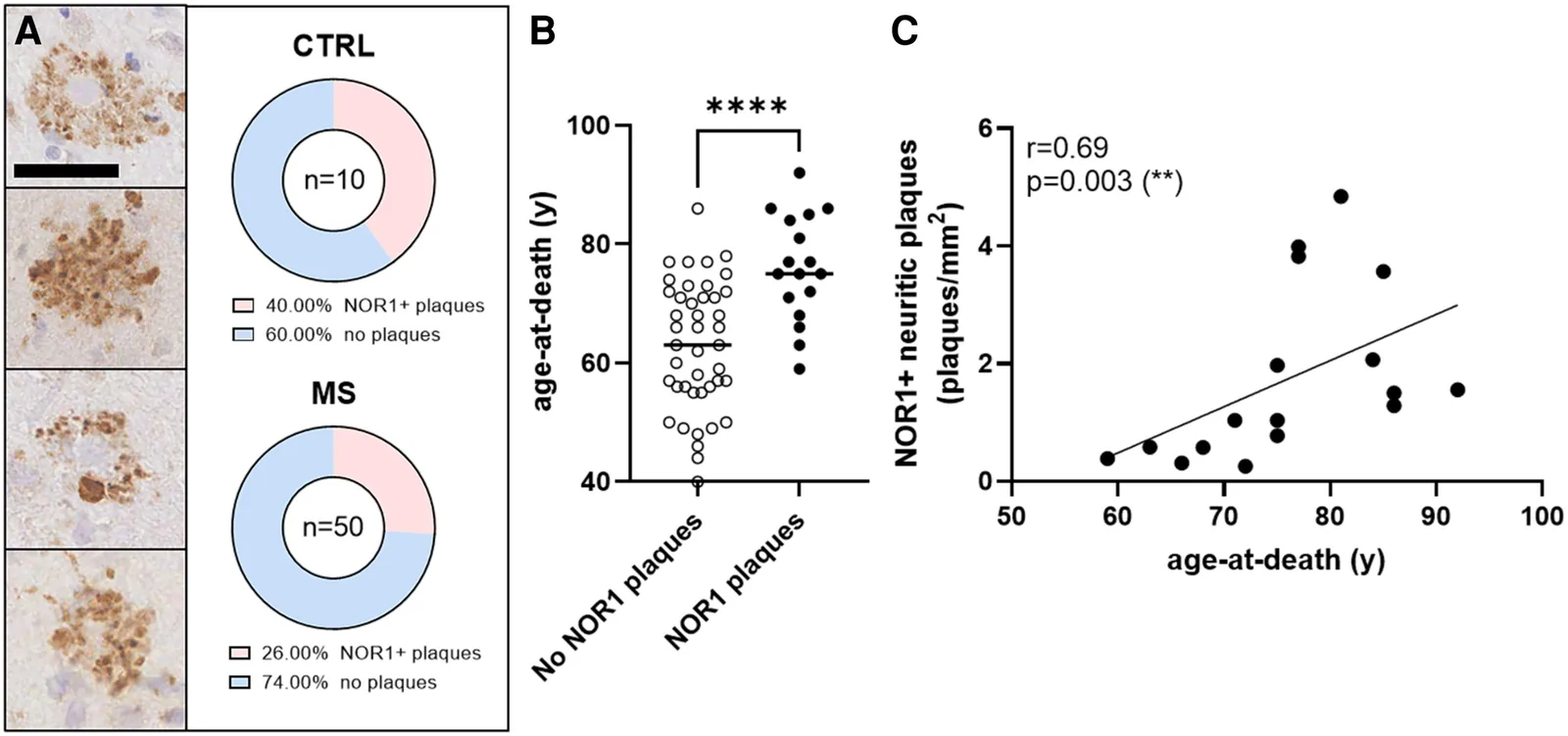
**NOR1+ neuritic profiles are related to ageing rather than being multiple sclerosis specific.** (**A**) Representative images of NOR1^+^ neuritic profiles. These neuritic profiles exhibit a round to ovoid morphology with vesicular or compact appearance. Accompanying pie charts indicate the proportion of multiple sclerosis (*n* = 50) and control cases (*n* = 10) displaying detectable NOR1^+^ neuritic profiles (blue: cases without neuritic profile; red: cases with neuritic profiles). (**B**) Older cases displayed more NOR1^+^ neuritic profiles than younger cases, regardless of disease status. (**C**) A positive correlation between age and the number of NOR1^+^ neuritic profiles per mm^2^ is observed across both multiple sclerosis and control cases, supporting that neuritic profile accumulation increases with ageing. (CTRL = control; Mann–Whitney U-test in (**B**), Spearman correlation in (**C**), **P < 0.01; *****P < 0.0001; NOR1 = neuron-derived orphan receptor 1: CTRL = control; in (**A**), brown colour represents NOR1-positive staining, purple colour represents haematoxylin counterstaining; each data-point represent the values for a single multiple sclerosis or control case which was positive for NOR1 neuritic profiles; in (**C**), each data-point represent the values for a single multiple sclerosis or control case which was positive for NOR1 neuritic profiles).

We assessed the presence of neuritic profiles immunoreactive for NOR1 (termed NOR1+ neuritic profiles) to examine whether NOR1 accumulation might be related to ageing and/or amyloid pathology (Fig. 5).

NOR1+ neuritic profiles were detected in both multiple sclerosis and controls, with a tendency towards lower prevalence in multiple sclerosis compared with controls (Fig. 5A). While this observation aligns with our previously published findings of reduced cortical Aβ plaque burden in multiple sclerosis,^25^ it should be noted that multiple sclerosis cases were younger than controls. Consistent with this, the presence and density of NOR1+ neuritic profiles in cases showing this pattern strongly correlated with age-at-death in our total cohort (multiple sclerosis and controls combined, Fig. 5B and C), but did not associate independently with disease status.

Of note, although most of NOR1+ neuritic profiles were spatially associated with 4G8+ amyloid plaques, NOR1 immunoreactivity was also observed in the absence of detectable Aβ deposition (data not shown), suggesting that NOR1 accumulation is not strictly restricted to classical amyloid pathology. Furthermore, AT8+ neuritic plaques were not detected in this cohort, precluding assessment of phospho-tau co-localization.

### NOR1 loss relates to biological ageing in multiple sclerosis motor cortex

**Figure 6:**
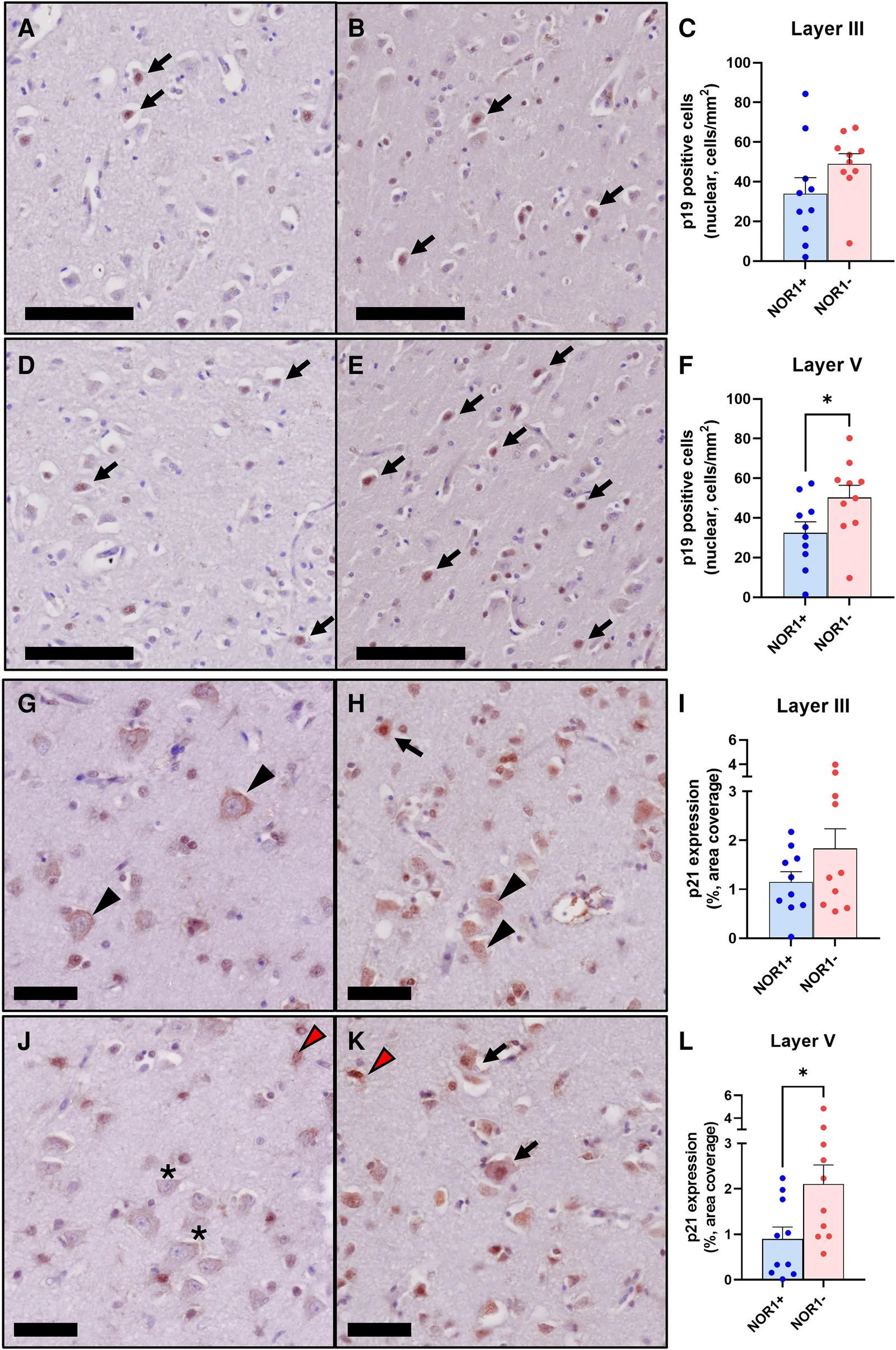
**NOR1 loss in layer V is associated with increased senescence markers in multiple sclerosis motor cortex.** (**A and B**) Representative images of p19 staining in layer III of (**A**) NOR1-high (*n* = 10) and (**B**) NOR1-low (*n* = 10) multiple sclerosis cases, with no significant difference in p19 expression (**C**). (**D and E**) Representative images of p19 staining in layer V of (**D**) NOR1-high and (**E**) NOR1-low multiple sclerosis cases, where p19 was significantly increased in NOR1-low cases compared with NOR1-high cases (**F**). (**G and H**) Representative images of p21 staining in layer III of (**G**) NOR1-high and (**H**) NOR1-low cases, with no significant difference observed (**I**). (**J and K**) Representative images of p21 staining in layer V of NOR1-high and NOR1-low cases, where p21 was significantly increased in NOR1-low cases (**L**). (Mann–Whitney U-test; *P < 0.05; NOR1 = neuron-derived orphan receptor 1; p19 = p19^INK4d; p21 = p21^CIP1; Arrows indicate nuclear p19^+^ and p21^+^ cells; black arrowheads indicate cytoplasmic p19^+^ neurons; red arrowheads indicate p19^+^ glial cells; stars indicate negative neurons; Scale bars 50 µm; in (**A, B, D and E**) brown colour represents p19-positive staining, purple colour represents haematoxylin counterstaining; in (**G, H, J and K**) brown colour represents p21-positive staining, purple colour represents haematoxylin counterstaining; in (**C, F, I and L**), each data point represents the values for a single multiple sclerosis case at the extremes of NOR1 expression).

To determine whether loss of NOR1 contributes to biological ageing processes in the multiple sclerosis motor cortex, we examined the expression of key senescence markers, p19 and p21, focusing on multiple sclerosis cases at the extremes of NOR1 expression (Fig. 6).

The p19 expression displayed predominant nuclear staining in pyramidal neurons and other brain cells, potentially interneurons and glial cells. No differences were detected in layer III between cases with low and high NOR1 expression, while p19 was increased in layer V of NOR1-low compared with NOR1-high cases (Fig. 6A–F; cells/mm^2^, layer III, NOR1-high: 34.0, CI95% 15.7–52.2 versus NOR1-low: 49.0, CI95% 37.3–60.7; *P* = 0.07; layer V, NOR1-high: 32.5, CI95% 19.9–45.1 versus NOR1-low: 50.3, CI95% 36.4–64.3; *P* = 0.028).

For p21, expression was mainly cytoplasmic, but in NOR1-low cases, we also observed a subset of neurons with nuclear p21 localization, a feature linked to deleterious senescent signalling.^26^ Like p19 expression, p21 was increased in layer V of NOR1-low compared with NOR1-high cases, whereas no differences were detected in layer III (Fig. 6G–L; area coverage in %, layer III, NOR1-high: 1.1, CI95% 0.7–1.6 versus NOR1-low: 1.8, CI95% 0.9–2.7; *P* = 0.43; layer V, NOR1-high: 0.9, CI95% 0.3–1.5 versus NOR1-low: 2.1, CI95% 1.1–3.0; *P* = 0.03).

## Discussion

In this study, we investigated NOR1 expression in the multiple sclerosis and control motor cortex and found a clear laminar divergence. NOR1 was preserved in layer III, where it was associated with higher neuronal density and Nurr1 expression, suggesting a conserved protective role. In contrast, NOR1 was reduced in layer V, where its loss correlated with increased glial activation and elevated senescence markers (p19, p21), pointing to a role in cortical vulnerability.

NOR1 expression in layer III was positively associated with both neuronal density and Nurr1 expression. We previously reported a protective role for Nurr1 in the multiple sclerosis motor cortex, with associations to higher neuronal density and reduced inflammation in layer III.^14^ The co-occurrence of NOR1 and Nurr1 in supragranular neurons suggests that an NR4A transcriptional network may remain functional in these layers, contributing to the relative preservation of neurons. Transcription factors from the same family commonly engage in intertwined regulatory mechanisms, and comparable co-regulation of NR4A members has been described, where they coordinate synaptic activity and metabolic stress responses.^12,27,28^ The absence of such a relationship in layer V of multiple sclerosis cortex underscores a laminar divergence, with supragranular neurons retaining protective transcriptional programmes while infragranular neurons lose this adaptive capacity.

The selective reduction of NOR1 in infragranular layers aligns with the well-recognized vulnerability of layer V pyramidal neurons in progressive multiple sclerosis.^24,29,30^ These neurons are critical for corticospinal output, making them disproportionately prone to damage under chronic inflammatory stress. This highlights a broader principle of selective neuronal vulnerability: large projection neurons, such as layer V pyramidal neurons, hippocampal CA1 neurons in AD^31,32^ or substantia nigra dopaminergic neurons in Parkinson’s,^33^ sustain long axons and high synaptic activity, requiring intense metabolic support and sustained transcriptional programmes to maintain excitability and plasticity.^34^ In line with this, NOR1 has been shown in neurons exposed to excitotoxic and oxidative stress to regulate protective transcriptional programmes that enhance survival^12^ and, more broadly, to control stress-adaptation and anti-apoptotic pathways.^15,35^ Its loss in layer V neurons may therefore compromise intrinsic resilience to metabolic and inflammatory insults often seen in multiple sclerosis.

Conversely, we detected a selective increase of nuclear NOR1+ neurons in layer V in multiple sclerosis, correlating with neuronal density. This suggests that neurons retaining or translocating NOR1 into the nucleus are those able to mount a stress-adaptive response. Indeed, NR4A receptors, including NOR1, are known to translocate to the nucleus upon inflammatory or oxidative stimuli to initiate protective transcriptional programmes that limit apoptosis and metabolic collapse.^12,15^ This pattern implies a dual state in vulnerable deep-layer neurons: widespread loss of cytoplasmic NOR1 and overall downregulation marking vulnerability, coexisting with nuclear accumulation in a subset of surviving neurons. These findings reinforce the view that layer V neurons are particularly at risk, with NOR1 nuclear mobilization representing a late and/or insufficient adaptive mechanism in progressive multiple sclerosis.

We also observed NOR1 expression in vascular-associated cells, including endothelial- and pericyte-like profiles. This finding is consistent with reports from vascular biology, where NOR1 is induced by oxidative and inflammatory stress in endothelial and smooth muscle cells, and is implicated in pathological vascular remodelling.^36^ The functional significance of vascular NOR1 expression in the CNS remains unclear, but it raises the possibility that NOR1 may also participate in blood–brain barrier regulation or vascular–glial interactions in multiple sclerosis. Although this lies beyond the scope of the present study, it warrants further investigation, as it could provide an additional link between vascular dysfunction and cortical pathology in progressive multiple sclerosis.

In addition, increasing evidence supports a central pathological role of cortical glial inflammation in progressive multiple sclerosis pathology.^37^ In our study, reduced NOR1 expression correlated with increased astrocytic GFAP and microglial CD68 expression, particularly in layer V, supporting a loss of anti-inflammatory function. While direct studies on NOR1 expression in glia are limited, other NR4A family members have been shown to repress NF-κB-driven inflammatory transcription,^38,39^ and restrain microglial and lymphocytic inflammation in an experimental autoimmune encephalomyelitis mouse model of multiple sclerosis and other neurodegenerative conditions.^40-42^ Thus, it is plausible that NOR1 downregulation similarly disinhibits glial inflammatory cascades in multiple sclerosis cortex, amplifying neuronal stress. Our findings are consistent with these observations and suggest that NOR1 may share similar functions in multiple sclerosis motor cortex, although this requires further investigation.

A striking observation was the association between NOR1 loss and senescence markers in layer V of the multiple sclerosis motor cortex. At the extremes of NOR1 expression, p19 displayed nuclear localization in pyramidal neurons and other cells and was significantly elevated in NOR1-low cases in layer V, while no differences were seen in layer III. For p21, expression was mostly cytoplasmic, but in NOR1-low cases a subset of neurons showed nuclear localization, a phenotype linked to irreversible cell cycle arrest and maladaptive stress signalling.^43,44^ Like p19, p21 was selectively increased in layer V of NOR1-low cases. Senescence pathways are increasingly recognized in the multiple sclerosis brain, with p16, p19 and p21 expression reported in both neurons and glia.^8,45^ Notably, p16 and p21 are key mediators of DNA damage responses and cell cycle checkpoint activation, and nuclear p21 in particular is closely linked to DNA damage-induced growth arrest in postmitotic neurons.^46-48^ In parallel, emerging evidence implicates NOR1 in the regulation of stress-responsive transcriptional programmes, including pathways involved in DNA repair and genomic stability. Thus, the concomitant reduction of NOR1 and increase of p19/p21 in vulnerable layer V neurons raises the possibility that impaired NOR1 signalling may compromise DNA damage handling, thereby favouring senescence-associated pathways under chronic inflammatory stress. Together, the parallel rise of p19 and p21 points to a coordinated activation of senescence and DNA damage responses in NOR1-deficient neurons in progressive multiple sclerosis, though further validation in neuronal systems is required.

Finally, NOR1+ neuritic profiles were identified in both multiple sclerosis and control cortices, strongly associated with ageing rather than disease status. Their frequent co-localization with amyloid is reminiscent of ubiquitin aggregates associated with amyloid plaques in Alzheimer’s,^49,50^ consistent with age-related proteostatic stress rather than disease-specific sequestration. Furthermore, their presence in areas lacking Aβ indicates that amyloid entrapment is not the sole explanation, and consequently does not contradict the previous findings of specific reduction of Aβ plaques in progressive multiple sclerosis.^25^ Alternative possibilities include extracellular release from degenerating neurons, impaired proteostasis and clearance with ageing, or accumulation in association with other ageing-related deposits. These mechanisms are not mutually exclusive and highlight the need for further investigation into NOR1+ neuritic profiles in the ageing brain and neurological conditions. Given the absence of AT8+ neuritic plaques in our cohort, structural characterization of these NOR1+ profiles would require complementary histochemical approaches, such as silver impregnation techniques to delineate dystrophic neurites and plaque architecture, combined with NOR1 and Aβ immunolabelling to clarify their intra- versus extracellular localization and relationship to amyloid pathology.

We acknowledge that this study has some limitations. As it relies on post-mortem tissue, it provides only a static snapshot of disease processes and cannot resolve temporal dynamics of NOR1 expression or its causal role in neuroinflammation and biological ageing. Control cases were also older than multiple sclerosis cases, which may influence ageing-related readouts (especially for p19 and p21), although our p19/p21 analyses were confined to multiple sclerosis cases to consistently point to disease-specific effects. In addition, detailed clinical histories were not consistently available across the cohort, as many cases date back to the 1990s and early 2000s. Therefore, useful stratification based on symptomatology (e.g. motor versus non-motor features) was unfortunately not feasible without introducing potential bias. We also acknowledge the unequal group sizes between multiple sclerosis and control cases, a common limitation in human post-mortem neuropathological studies,^2,5,24^ which we mitigated through rigorous statistical and methodological controls. Mechanistic insights also remain limited, as functional studies were beyond the scope of this work, and some interpretations draw on parallels with other NR4A family members, given the paucity of direct data on NOR1 in the CNS in general, and in multiple sclerosis in particular. Nonetheless, several features support the robustness of our conclusions: we analysed a large, well-characterized multiple sclerosis cohort with qualitative, quantitative and spatially resolved assessments, used multiple markers of neurons, glial cells and senescence, and validated antibody specificity with stringent controls. These strengths ensure that our findings reliably address the central question of whether NOR1 is altered in the cortex and how this relates to multiple sclerosis pathology.

In conclusion, our findings reveal that NOR1 loss relates to biological ageing and inflammation in the multiple sclerosis motor cortex in a layer-specific manner. Its preservation in superficial layer III may support neuronal integrity and Nurr1-associated protective programmes, whereas its loss in vulnerable layer V projection neurons coincides with glial activation and senescence. This laminar divergence highlights the importance of spatially resolved analyses in multiple sclerosis neuropathology and suggests that NOR1 is a previously unrecognized regulator of cortical resilience. While further work is needed to determine whether its loss is a cause or consequence of chronic neuroinflammatory stress, this study provides the first detailed mapping of NOR1 in the GM of the multiple sclerosis motor cortex and uniquely positions this transcription factor, alongside other NR4A family members, as a potential target to mitigate progressive cortical neurodegeneration and, by extension, the accumulation of irreversible disability in progressive multiple sclerosis.

## Supplementary Material

fcag315_Supplementary_Data

## Data Availability

The data that support the findings of this study are available from the corresponding author, upon reasonable request.
