# Supplementary material for: NOR1 loss relates to inflammation and biological ageing in multiple sclerosis motor cortex
Source: Brain Commun. 2026 Aug 14;8(4):fcag315. doi: 10.1093/braincomms/fcag315 (PMC13519354; doi:10.1093/braincomms/fcag315)
Supplement: Supplementary file 1 [file fcag315_supplementary_data.zip › Supplementary_material_Glossary.docx]

**Glossary**

**NOR1 loss relates to inflammation and biological ageing in multiple sclerosis motor cortex**

Buse Unlu Kobya^1^, Aimee Avery^1^, Gabriele DeLuca^1†^, Jonathan Pansieri^1†^

^†^These authors contributed equally to this work

NR4A = nuclear receptor subfamily 4A

NOR1 = neuron-derived orphan receptor 1

Nurr1 = Nuclear Receptor Related-1 Protein

Nur77 = Neuron-derived orphan receptor 77

GM = grey matter

MS = multiple sclerosis

GFAP = Glial fibrillary acidic protein

CD68 = Cluster of Differentiation 68

NeuN = Neuronal Nuclear Antigen

DAB = 3,3′-diaminobenzidine

IF = immunofluorescence

FOV = field of view

GLM = Generalized Linear Model

NLWM = non-lesional white matter

NLGM = non-lesional grey matter

PMI = post mortem interval

EAE = Experimental Autoimmune Encephalomyelitis
