# Supplementary material for: NOR1 loss relates to inflammation and biological ageing in multiple sclerosis motor cortex
Source: Brain Commun. 2026 Aug 14;8(4):fcag315. doi: 10.1093/braincomms/fcag315 (PMC13519354; doi:10.1093/braincomms/fcag315)
Supplement: Supplementary file 1 [file fcag315_supplementary_data.zip › Supplementary_Tables_and_Figures.pdf]

Supplementary information

NOR1 loss relates to inflammation and biological ageing in multiple sclerosis motor cortex

Buse Unlu Kobya[1], Aimee Avery[1], Gabriele DeLuca[1†], Jonathan Pansieri[1†]

[†]Jonathan Pansieri and Gabriele DeLuca contributed equally to this work

Author affiliations:

1 Nuffield Department of Clinical Neurosciences, University of Oxford, Level 6 West Wing, John Radcliffe Hospital, OX3 9DU Oxford, UK

Correspondence to: Jonathan Pansieri

Oxford University, Nuffield Department of Clinical Neurosciences, Oxford University. Level 1 Neuropathology, John Radcliffe Hospital, West Wing, Oxford, OX3 9DU, UK

E-mail : jonathan.pansieri@ndcn.ox.ac.uk

Correspondence may also be sent to: Gabriele Carmine De Luca

Oxford University, Nuffield Department of Clinical Neurosciences, Oxford University. Level 1 Neuropathology, John Radcliffe Hospital, West Wing, Oxford, OX3 9DU, UK

E-mail : gabriele.deluca@ndcn.ox.ac.uk

Running title: Reduction of NOR1 in Multiple Sclerosis motor cortex

Keywords: multiple sclerosis, ageing, inflammation, NR4A3

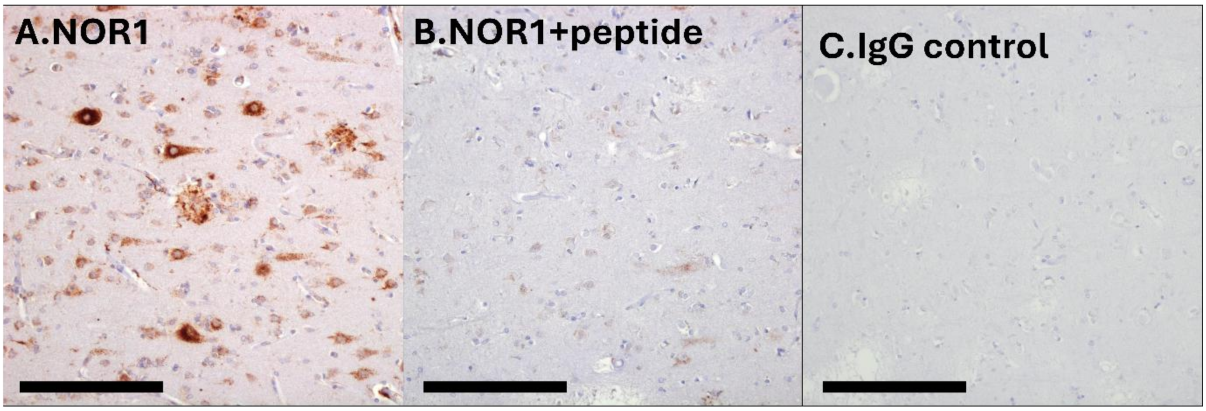

Supplementary Fig. 1. Validation of NOR1 immunohistochemistry specificity. (A) NOR1 immunoreactivity is observed in neurons and neuritic plaques in the multiple sclerosis motor cortex. (B) Pre-incubation of the primary antibody with a recombinant NOR1 control fragment (aa 377–455) markedly reduced the staining in the same multiple sclerosis case, confirming specificity. (C) Isotype-matched IgG control showed no staining in the same multiple sclerosis case. Scale bars: 100 µm. Brown colour represent positive staining, purple colour represent haematoxylin counterstaining.

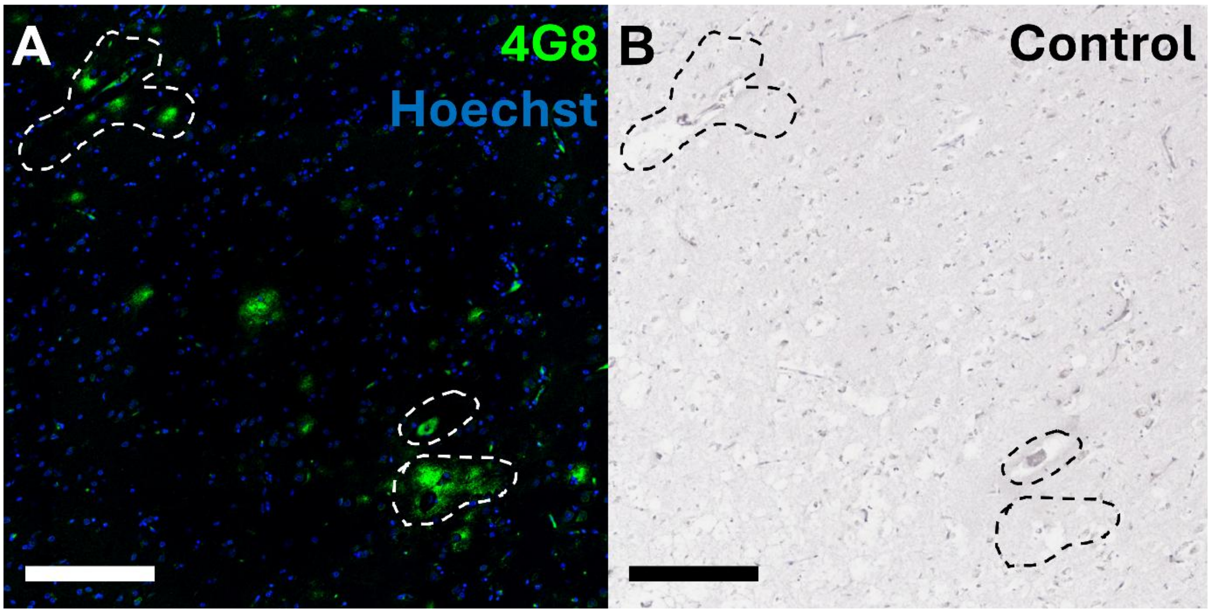

Supplementary Fig. 2. Control experiment showing successful stripping process. (A) Representative immunofluorescence picture of amyloid (green, 4G8) staining showing amyloid plaques in multiple sclerosis motor cortex, alongside nuclear staining (blue, Hoechst). **(B)** Control DAB staining after stripping process on the same section, showing absence of amyloid staining. Scale bar 200µm. In **(B),** purple/blue colour represent haematoxylin counterstaining.

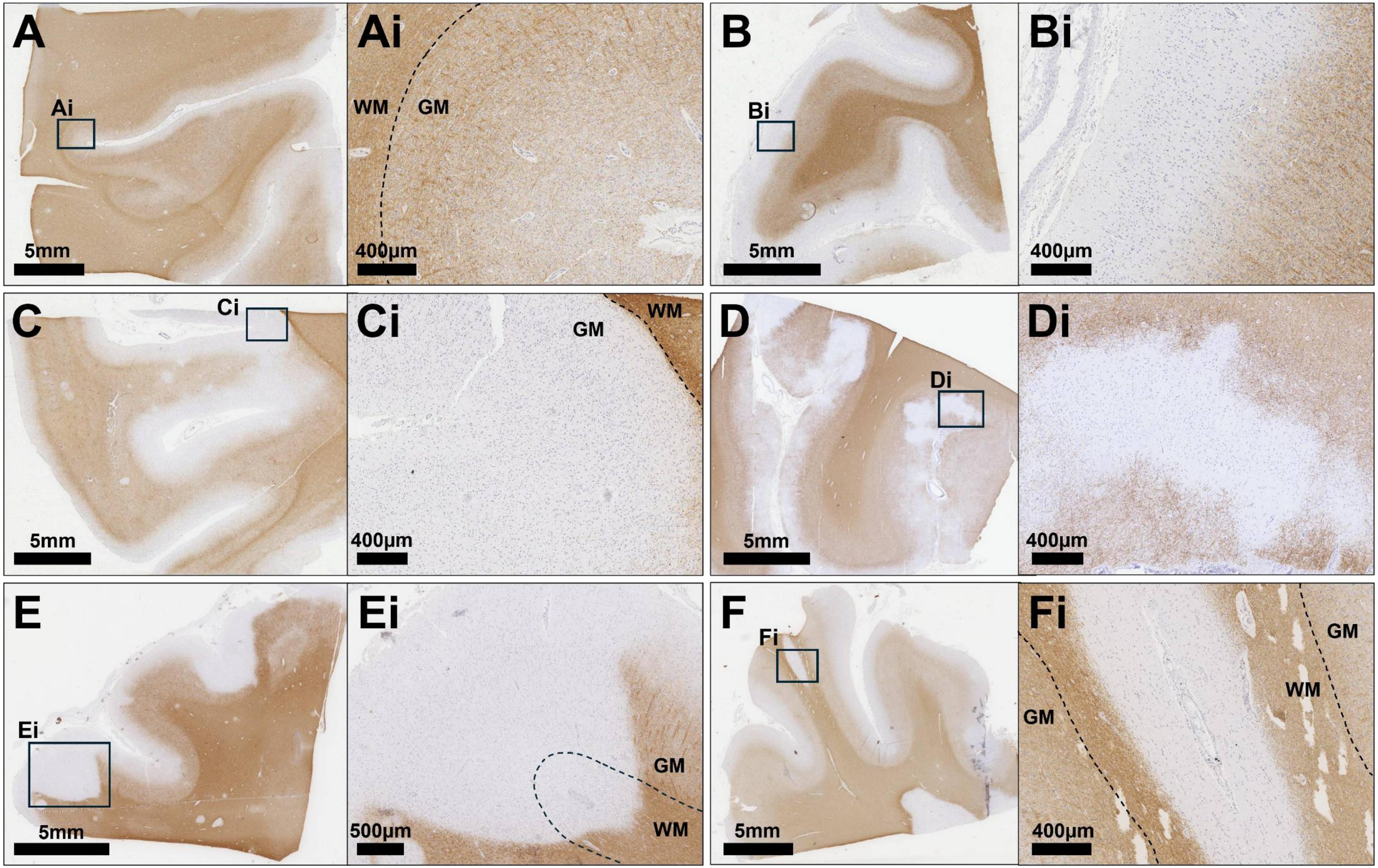

A
Ai
5mm
Ai
WM
GM
400µm
B
Bi
5mm
Bi
400µm
C
Ci
5mm
Ci
GM
WM
400µm
D
Di
5mm
Di
400µm
E
Ei
5mm
Ei
GM
WM
500µm
F
Fi
5mm
Fi
GM
WM
GM
400µm

Supplementary Fig. 3. Demyelination in MS motor cortex. Representative images of the motor cortex stained for proteolipid protein (PLP) illustrating areas of complete myelin loss. **(A, Ai)** Example of MS motor cortex with preserved myelin in both white and grey matter. **(B, Bi)** Subpial lesions are located in the superficial cortical layers, immediately beneath the pia mater, extending from the pial surface into the supragranular layers without involving infragranular layers. **(C, Ci)** Pan-cortical lesions resemble subpial lesions but extend deeper, involving both supra- and infragranular layers. **(D, Di)** Intracortical lesions are restricted to the cortical grey matter and do not extend to either the pial surface or the underlying white matter. **(E, Ei)** Leukocortical lesions involve both the subcortical white matter and the adjacent cortical grey matter. **(F, Fi)** White matter lesions are confined to the white matter and do not extend into the cortical grey matter. (Dotted lines indicate the boundary between white and grey matter ; WM = white matter ; GM = grey matter). . in **(A-F)** brown colour represent PLP positive staining, purple colour represent haematoxylin counterstaining.

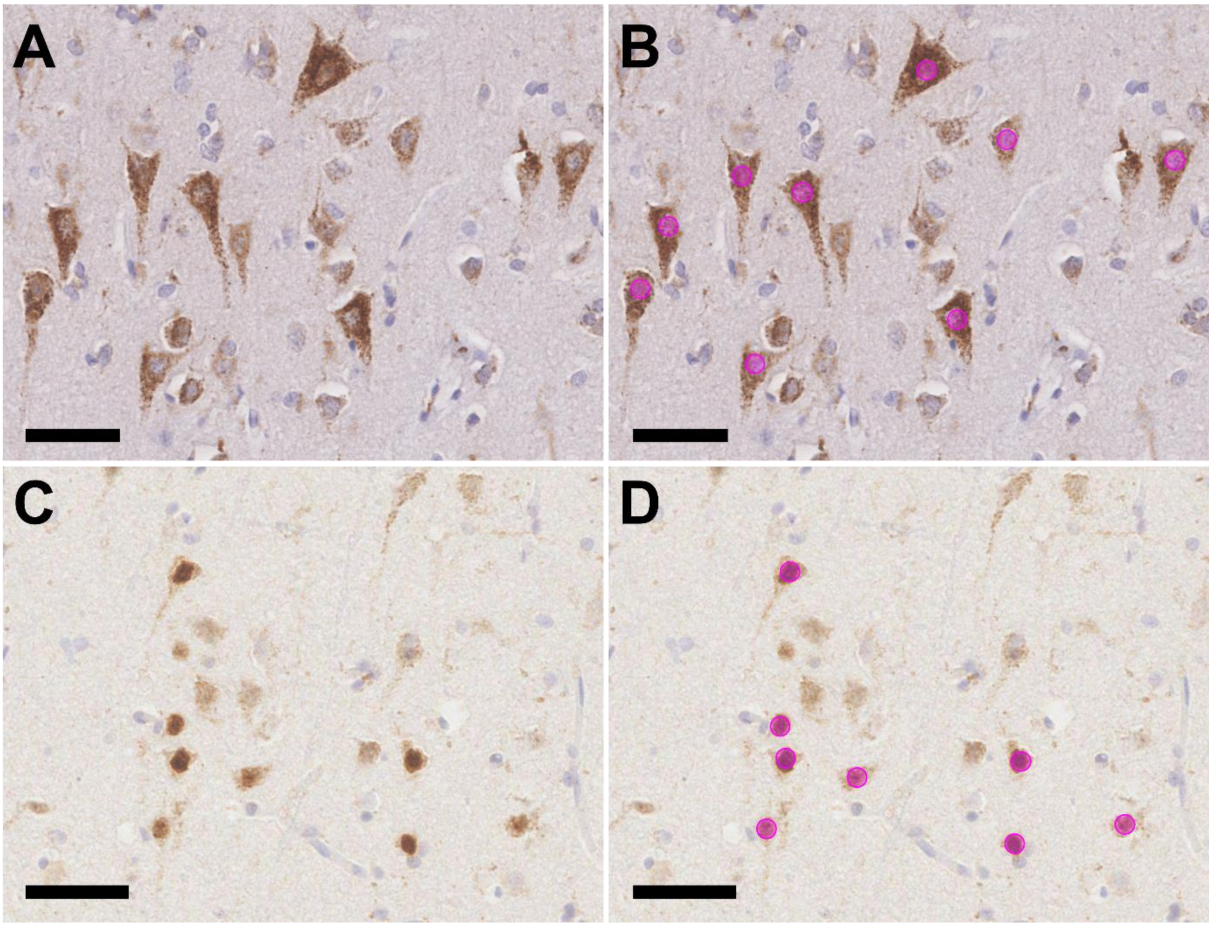

Supplementary Fig. 4. Quantification of NOR1⁺ neurons. (A) Representative image of NOR1 immunoreactivity in the cytoplasm of pyramidal neurons (layer III) of the multiple sclerosis motor cortex. **(B)** Example of manual annotation of NOR1⁺ neurons (highlighted in magenta) meeting inclusion criteria. **(C)** Representative image of NOR1 immunoreactivity in the nucleus of pyramidal neurons (layer V) of the multiple sclerosis motor cortex. **(D)** Example of manual annotation of NOR1⁺ neurons (highlighted in magenta) meeting inclusion criteria. Scale bars 50µm. Brown colour represent NOR1 positive staining, purple colour represent haematoxylin counterstaining.

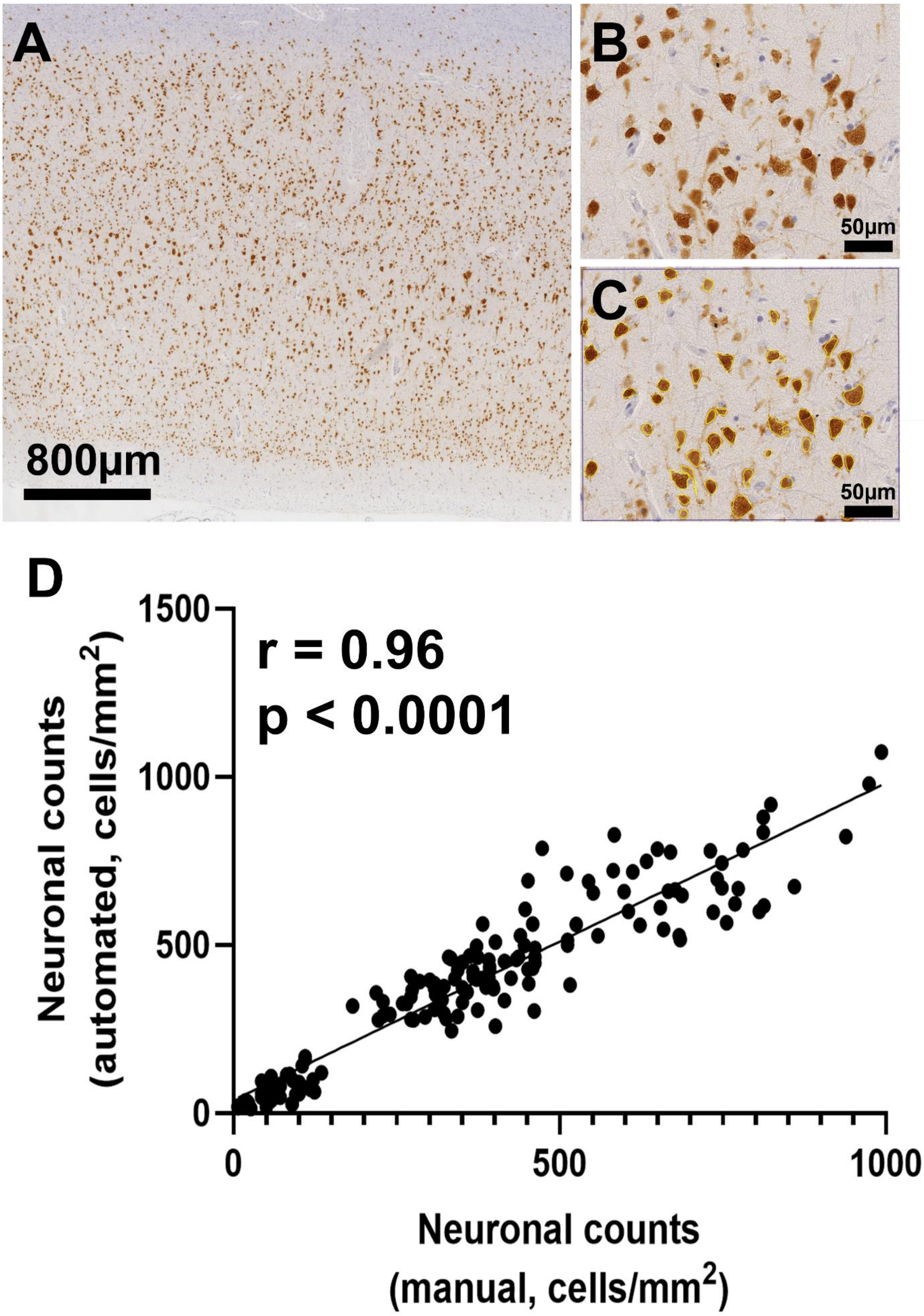

Supplementary Fig. 5. Validation of automated neuronal density quantification. (A) Representative low-magnification picture of NeuN staining in the MS motor cortex. **(B)** High-

magnification view showing NeuN-positive neurons. **(C)** Example of automated detection of NeuN+ neurons using QuPath (yellow outlines) in layer III. **(D)** Correlation between manual and automated neuronal counts of $NeuN^+$ neurons per field of view (FOV) in cortical layers III and V. Each dot represents one FOV; three cases from our cohort were analysed. Automated quantification using QuPath correlated with manual counts (Spearman correlation, $r = 0.96$, $p < 0.0001$), confirming the reliability of the automated approach. In **(A-C),** brown colour represent NeuN positive staining, purple colour represent haematoxylin counterstaining. in D, each data-point represent a single annotation value.

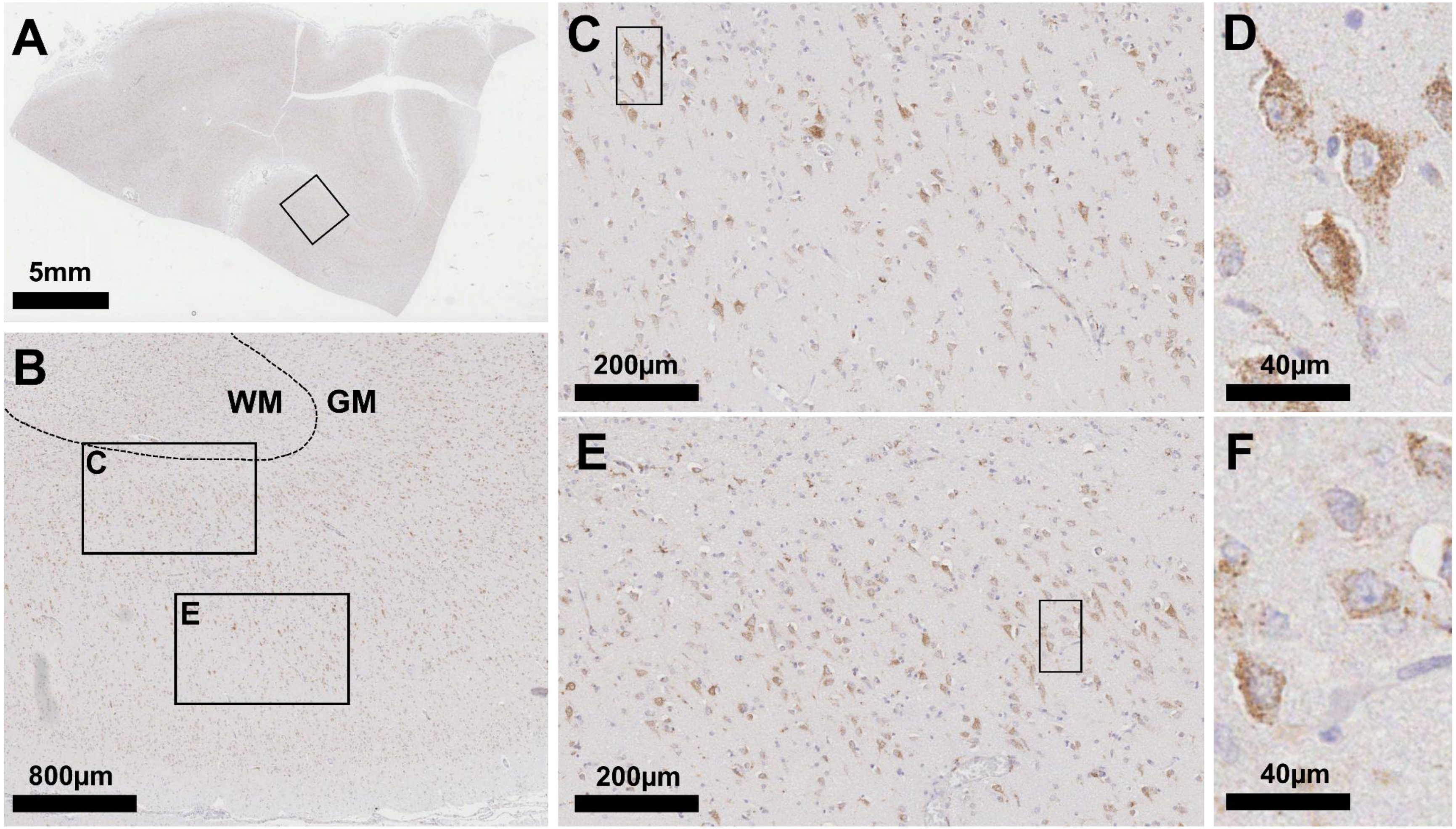

Supplementary Fig. 6. Representative pictures of NOR1 extent in the motor cortex. (A) Low-magnification overview of control motor cortex stained with NOR1 indicating the region of interest in B. **(B)** Higher-magnification view showing the boundary between white matter (WM) and the grey matter cortical ribbon grey matter (GM), with selected magnified areas in C and E. **(C-F)** Representative NOR1 staining in **(C)** layer III

and **(E**) layer V of the motor cortex with respective **(D, F)** High-magnification insets showing NOR1 expression and highlighting predominant cytoplasmic neuronal patterns. Brown colour represent NOR1 positive staining, purple colour represent haematoxylin counterstaining.

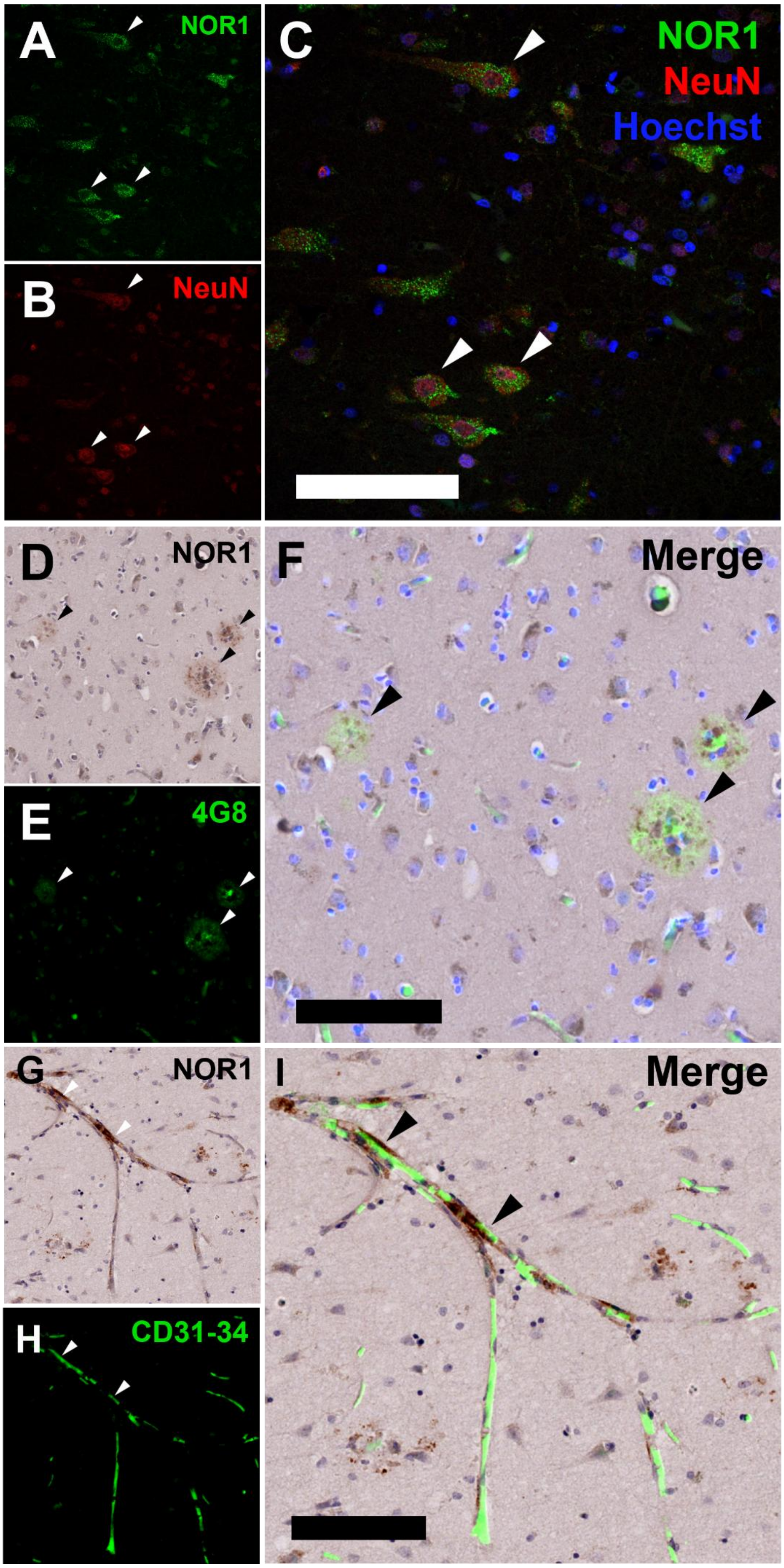
A
NOR1
B
NeuN
C
NOR1
NeuN
Hoechst
D
NOR1
E
4G8
F
Merge
G
NOR1
H
CD31-34
I
Merge

Supplementary Fig. 7. NOR1 is predominantly expressed in neurons, but also show a plaque-associated profile in multiple sclerosis and control motor cortex. Representative pictures using IF and IHC, showing that **(A-C)** NOR1 (green) is mainly expressed in the cytoplasm of layer III and layer V pyramidal neurons (NeuN, red). **(D-F)** NOR1 (IHC, brown) is sparsely expressed in endothelial cells (CD31-34, green) and **(G-I)** co-locates with amyloid plaques (4G8 staining, green).(All merge pictures are supplemented by nuclear staining using Hoechst excepting **(G-I)** ; arrowheads highlight co-labelling of interest ; scale bars 100µm. IF=immunofluorescence ; IHC=immunohistochemistry)

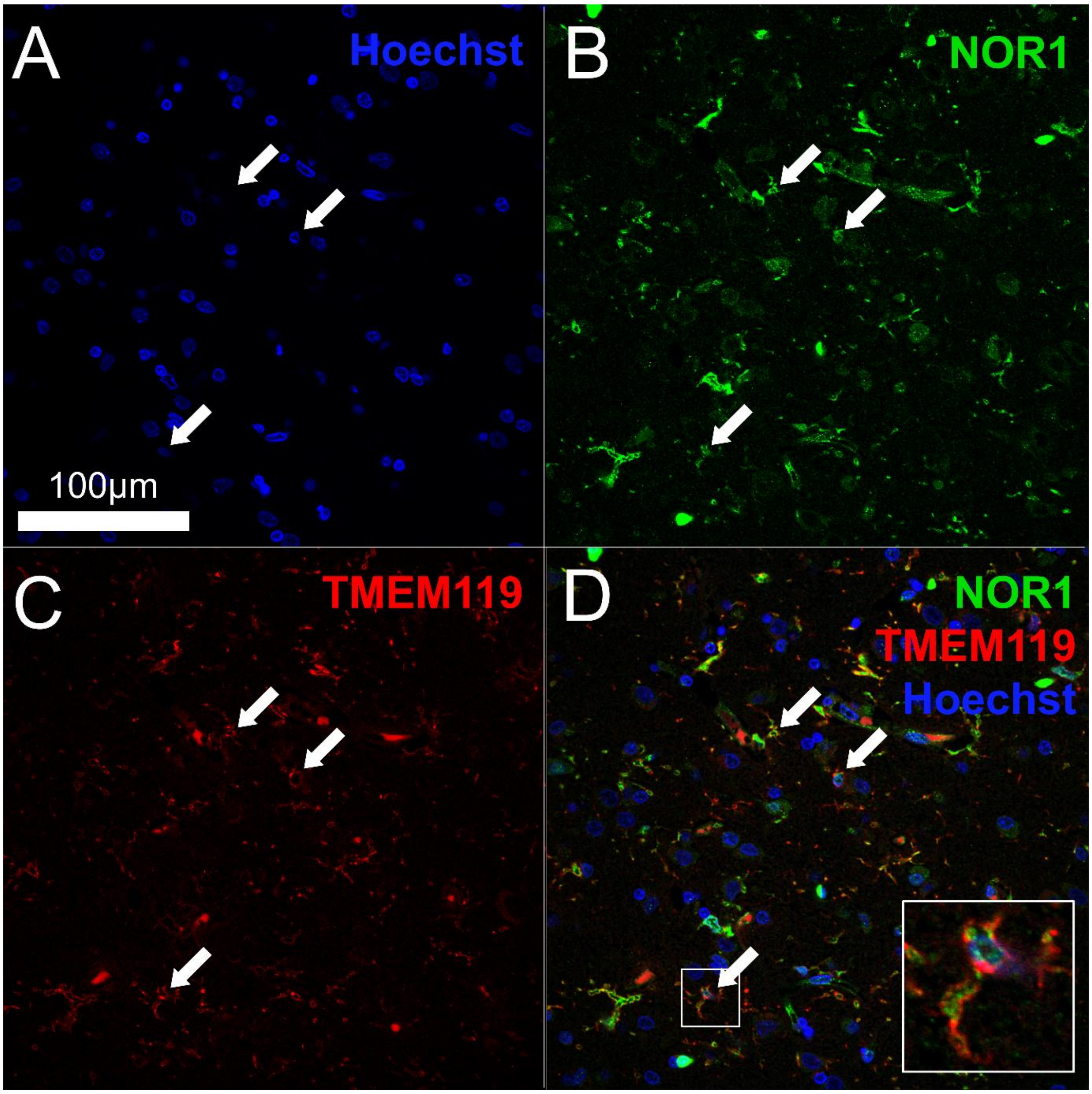

Supplementary Fig. 8. NOR1 is rarely found in microglial cells in some multiple sclerosis and control motor cortices. (A) Nuclear staining (Hoechst) was added to **(B)** NOR1 (green), which is rarely found in **(C-D)** microglia (TMEM119, red). (Merge picture in **(D)** ; arrowheads highlight co-labelling of interest ; magnified picture in **(C)** shows a Hoechst+TMEM119+NOR1+ microglia. (400X magnification).

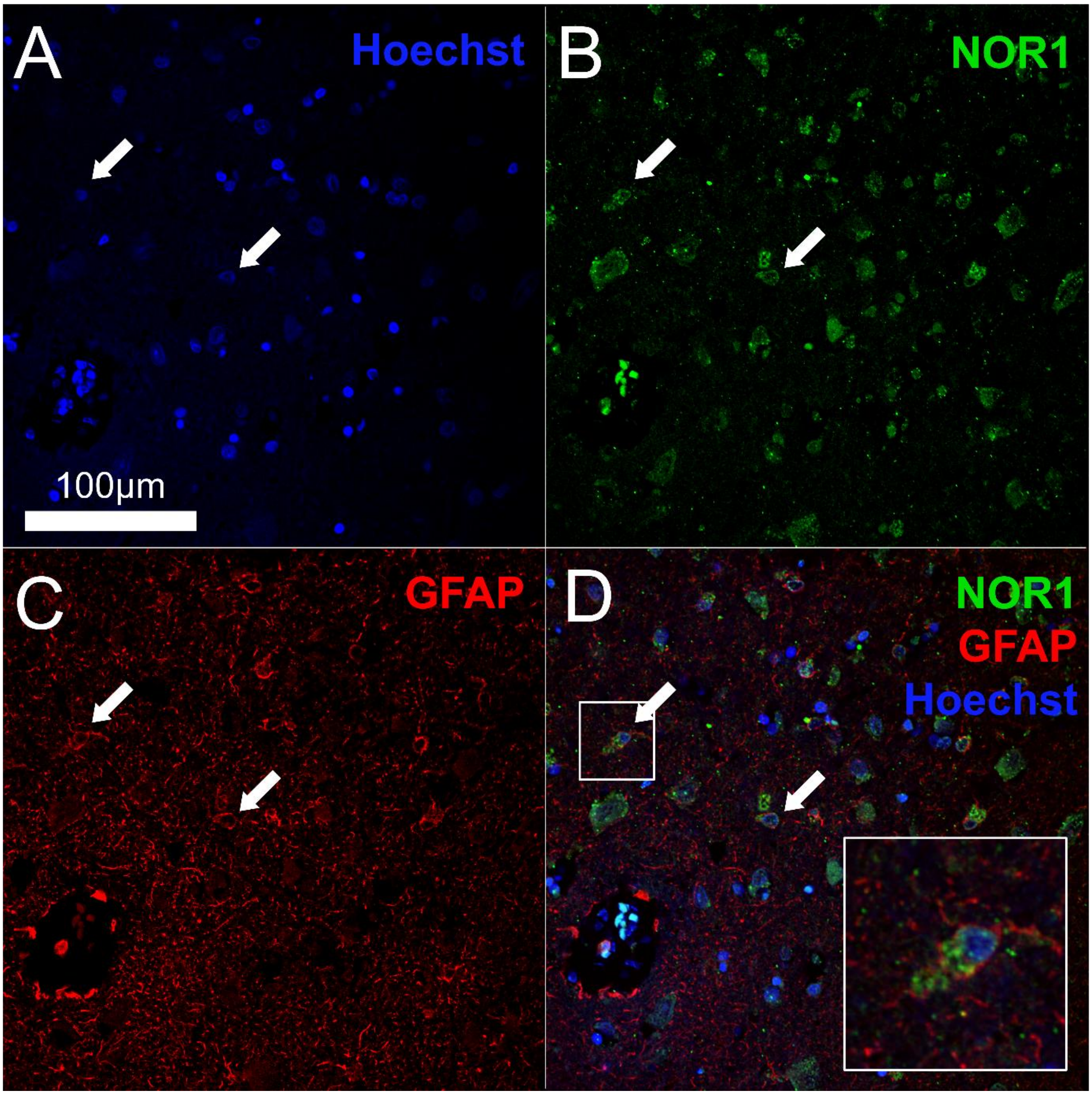

Supplementary Fig. 9. NOR1 is rarely expressed in astrocytes in some multiple sclerosis and control motor cortices. (A) Nuclear staining (Hoechst) was added to **(B)** NOR1 (green), which is rarely found in **(C)** astrocytes (GFAP, red). (merge picture in **(D)** ; arrows highlight co-labelling of interest ; magnified picture in **(D)** shows a Hoechst+GFAP+NOR1+ astrocyte. (400X magnification)

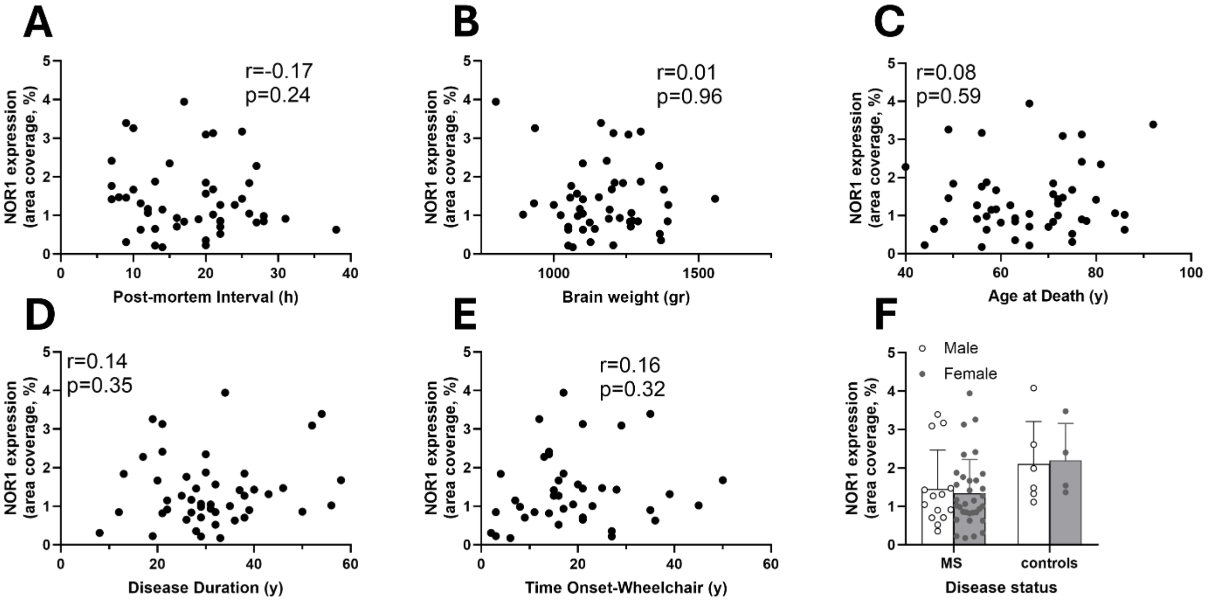

Supplementary Fig. 10. Relationships between NOR1 expression in non-lesional grey matter and clinical features in multiple sclerosis. Spearman correlation analyses showing no significant relationship between total NOR1 expression in the non-lesional grey matter and **(A)** post-mortem interval, **(B)** brain weight, **(C)** age at death, **(D)** disease duration, **(E)** time from onset to wheelchair in multiple sclerosis cases and (all comparisons P > 0.9). **(F)** Sex-based comparisons in both multiple sclerosis and control cases, respectively, show no difference in NOR1 expression comparing male versus female subgroups in the non-lesional grey matter (both comparisons P > 0.9). Of note, adding PMI or age-at-death as covariates did not impact the results (generalized linear model). (Spearman correlation analysis in **A-E**, Mann-Whitney U-test comparing male and female subgroups in both MS and control cases respectively in **F**). in **(A-E),** each data-point represent the values for a single MS case. in **(F),** each data-point represent the values for a single MS or control case where appropriate.

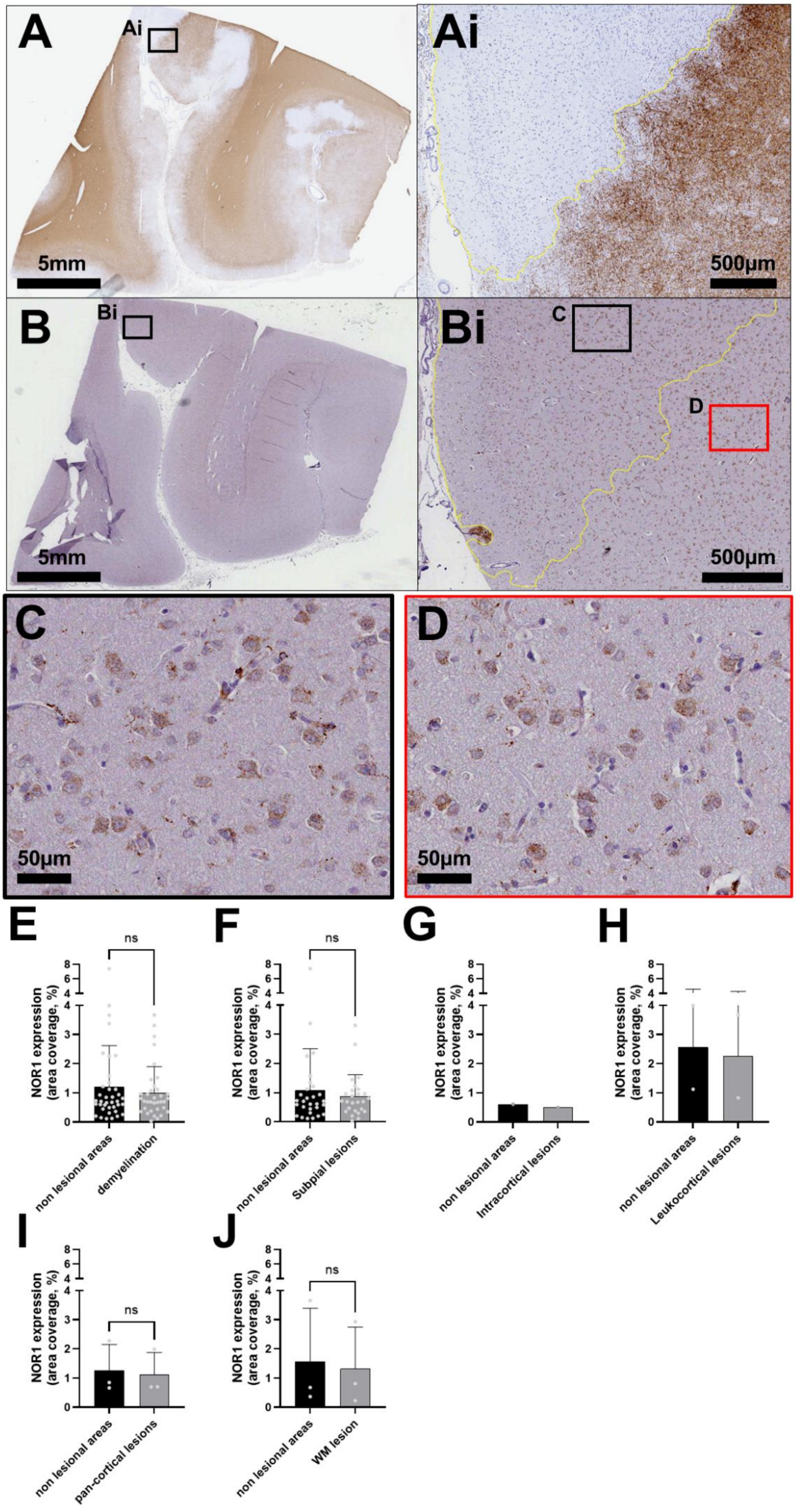
A
Ai
5mm
500µm
B
Bi
C
D
50µm
E
F
G
H
I
J
ns
NOR1 expression (area coverage, %)
non lesional areas
demyelination
Subpial lesions
Intracortical lesions
Leukocortical lesions
pan-cortical lesions
WM lesion

Supplementary Fig. 11. NOR1 expression does not relate to demyelination in MS motor cortex. NOR1 immunoreactivity was assessed in lesional areas compared to non-lesional areas. **(A)** Representative low-magnification image of a subpial demyelinated cortical region (PLP staining) with corresponding higher-magnification view **(Ai),** illustrating the lesion boundary (yellow). **(B)** Adjacent section stained to assess NOR1 expression, with higher-magnification view (Bi). Representative NOR1 staining in **(C)** lesional and **(D)** non-lesional areas, showing no evident differences in NOR1 expression. Quantitative comparisons across **(E)** all demyelinated areas combined (n=35) and **(F–J)** individual demyelinated lesion subtypes, namely subpial (n=26), intracortical (n=1), leukocortical (n=2), pancortical (n=3) and WM lesion (n=3) and their anatomically matched non-lesional regions, demonstrated no significant differences in NOR1 expression (all comparisons, $P > 0.50$). Only lesions for which a corresponding anatomically matched non-lesional region was available were included in the quantitative analyses. For subtype-specific analyses **(F–J),** when multiple lesions of the same subtype were present within a case, lesion values were averaged prior to statistical analysis such that each data point represents a single multiple sclerosis case. Statistical comparisons in **(F-J)** were performed using the Wilcoxon matched-pairs signed-rank test. For **(E),** because a single case could contribute more than one lesion subtype, statistical analysis was performed using a linear mixed-effects model, with lesion status (lesional versus matched non-lesional) included as a fixed effect and multiple sclerosis case as a random effect to account for within-case clustering. Data are shown as mean ± SEM ; ns denotes non-significant differences. in **(A, Ai)** brown colour represent PLP positive staining, purple colour represent haematoxylin counterstaining. In **(B-D)** brown colour represent NOR1 positive staining, purple colour represent haematoxylin counterstaining. In **(E-J),** each data-point represent the values for a lesional or anatomically matched non-lesional area in multiple sclerosis cases when appropriate.

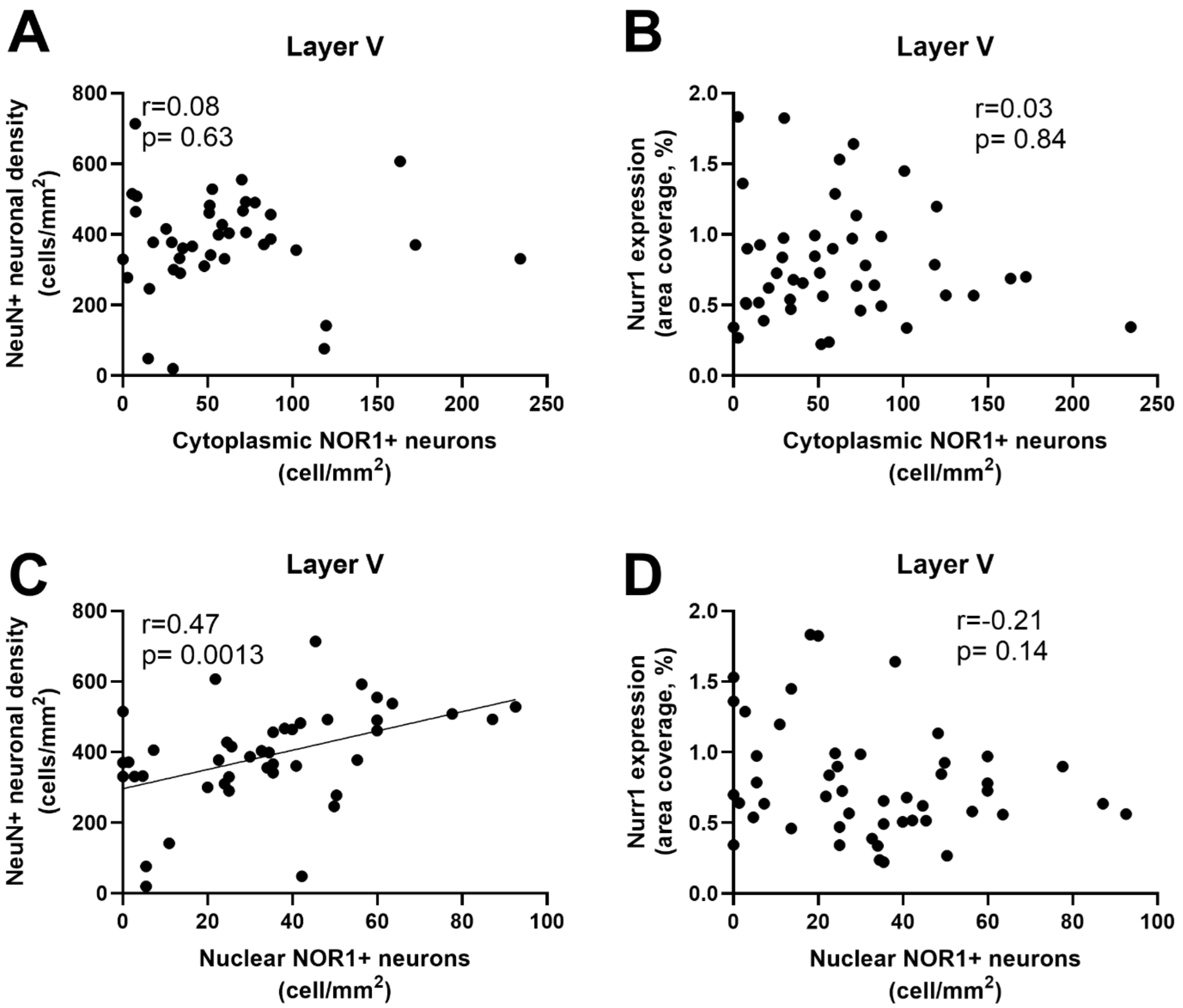

Supplementary Fig. 12. Relationships between sub-cellular NOR1 and Nurr1 or neuronal density in layer V in multiple sclerosis motor cortex. Correlation analyses showing no significant relationship between cytoplasmic NOR1+ neuronal density and **(A)** neuronal density or **(B)** Nurr1 expression. **(C)** A positive relationship between cytoplasmic NOR1+ neuronal density and neuronal density was found (p=0.47, p=0.0013) but not with **(D)** Nurr1 expression. (Spearman correlation analysis). Each data-point represent the values for a single MS case.

MS cases

ID	Age (y)	Sex	MS subtype	DD (y)	TOW (y)	BW (gr)	PMI (h)	Cause of Death	PM diagnosis
**MS022**	57	F	SPMS	17	10	1050	38	Pneumonia, MS	Chronic MS
**MS047**	66	F	SPMS	34	17	800	17	MS	Chronic MS
**MS048**	84	F	SPMS	29	Unknown	1268	12	Bronchopneumonia, Cerebral Vascular Aneurysm, MS	Subarachnoid haemorrhage
**MS053**	66	M	SPMS	31	19	1100	26	Bronchopneumonia	Chronic MS
**MS062**	49	F	SPMS	19	12	935	10	Respiratory Infection	Chronic MS
**MS063**	66	F	SPMS	29	27	1050	13	Aspiration Pneumonia, MS, Peripheral Vascular Disease	Chronic MS. Generalised hypoxic changes.
**MS066**	86	F	SPMS	56	45	894	21	Pneumonia	Chronic MS
**MS067**	86	F	SPMS	36	36	1101	11	Bronchopneumonia	significant atherosclerosis. Demyelination in one corticospinal tract
**MS070**	77	F	PPMS	21	21	1206	21	MS	Chronic MS. Amyloid plaques in the cortex
**MS077**	57	F	PPMS	29	8	1083	28	Bronchopneumonia, MS	Chronic MS
**MS082**	49	F	SPMS	28	21	1057	9	Aspiration Pneumonia	Chronic MS
**MS086**	81	F	RRMS or SPMS	30	14	1100	15	Bladder Cancer, Chest Infection, MS	Chronic MS with potential AD type pathology
**MS089**	71	F	SPMS	27	Unknown	1263	17	Intercerebral Bleeding, MS	Chronic MS. Diffuse hypoxic changes
**MS097**	55	M	SPMS	22	Unknown	1190	31	Bronchopneumonia, MS	Chronic MS
**MS102**	73	M	PPMS	52	29	1258	20	Left Ventricular Failure, Pneumonia, Pleural Effusion	Chronic MS. Diffuse hypoxic changes
**MS103**	77	F	SPMS	21	14	1183	27	Pneumonia	Chronic MS
**MS105**	73	M	SPMS	46	25	1156	28	Pneumonia, Infection	Chronic MS
**MS111**	92	M	PPMS	54	35	1163	29	Old Age	Chronic MS. Demyelination of a mild degre
**MS115**	75	F	SPMS	58	50	1200	21	Not Reported	no evidence of demyelination.
**MS120**	72	F	SPMS	35	23	1024	21	Metastatic Liver Disease, MS	Chronic MS
**MS126**	75	M	SPMS	32	16	1366	22	Pneumonia	Chronic MS
**MS128**	78	F	SPMS	50	Unknown	1272	22	Small Bowel Obstruction, Pneumonia	Chronic MS
**MS133**	63	M	SPMS	28	27	1370	20	Metastatic Lung Carcinoma, MS	Chronic MS. Features suggestive of cerebellar degeneration
**MS146**	63	F	SPMS	31	17	1228	16	Pneumonia, MS	Chronic MS
**MS147**	60	F	SPMS	21	14	1124	27	Pneumonia, MS	Chronic MS
**MS159**	55	F	SPMS	25	16	1000	24	Amitriptyline Overdose	Chronic MS
**MS162**	58	F	SPMS	22	7	1193	14	gastrointestinal Bleed from Ulcer, MS	Chronic MS

ID	Age (y)	Sex	MS subtype	DD (y)	TOW (y)	BW (gr)	PMI (h)	Cause of Death	PM diagnosis
**MS165**	59	F	SPMS	20	16	1380	10	Respiratory Failure, Cardiac Arrest	Chronic MS
**MS170**	56	M	SPMS	>28	>5	1300	25	Pancreatic Cancer	Chronic MS
**MS181**	71	F	SPMS	32	20	1080	20	Pneumonia, MS	Chronic MS
**MS182**	56	F	PPMS	33	6	1066	14	Aspiration Pneumonia, Sepsis	Chronic MS. Atherosclerotic changes
**MS187**	57	F	SPMS	30	Unknown	1300	13	MI, Ischemic Heart Disease, Sepsis, MS	Chronic MS
**MS191**	48	F	SPMS	32	3	1393	28	Pneumonia, MS	Chronic MS
**MS200**	44	F	SPMS	19	3	1205	20	UTI, Sepsis, MS	Chronic MS
**MS218**	56	F	SPMS	26	Unknown	1060	7	Chest Infection, Disseminated Multiple Myeloma, MS	Chronic MS
**MS222**	70	M	SPMS	38	9	1050	22	Bronchopneumonia, MS	Chronic MS
**MS231**	59	F	PPMS	27	Never	1090	12	Bronchopneumonia, MS	No evidence of demyelination
**MS237**	77	M	SPMS	39	35	1190	19	Pneumonia	Chronic MS
**MS266**	75	F	PPMS	8	2	1127	19	MS	Pathological features of Tauopathy. No evidence of demyelination
**MS269**	72	M	SPMS	40	28	1557	25	Bronchopneumonia, MS	Glioblastoma multiforme (grade IV) - Chronic MS
**MS298**	72	M	SPMS	43	39	932	11	Chest infection, MS	Chronic MS
**MS312**	68	F	SPMS	23	8	1150	24	Urinary Tract Infection, Sepsis, MS	Chronic MS
**MS313**	66	M	PPMS	29	21	1266	16	Gastrointestinal bleeding caused by peptic ulcer disease, MS	Chronic MS
**MS319**	63	F	SPMS	12	11	1291	22	Metastatic Bladder Carcinoma	Chronic MS
**MS335**	62	M	SPMS	38	15	1395	22	Aspiration Pneumonia, Renal Failure, MS	Chronic MS
**MS338**	50	F	SPMS	13	4	1239	26	Pulmonary Hemorrhage, MS	Chronic MS
**MS348**	46	F	SPMS	26	21	1142	13	MS	Severe chronic MS
**MS371**	40	M	SPMS	17	13	1364	27	Bronchopneumonia	Chronic MS with severe demyelination
**MS374**	71	F	SPMS	38	17	1210	20	Pneumonia	Chronic MS
**MS381**	80	F	SPMS	37	15	1101	7	MS	Chronic MS. AD-like age-related pathology
**Controls**									
**ID**	**Age (y)**	**Sex**	**MS subtype**	**DD (y)**	**TOW (y)**	**BW (gr)**	**PMI (h)**	**Cause of Death**	**PM diagnosis**
**C026**	**78**	**M**	N/A	N/A	N/A	**1250**	**33**	Myeloid Leukemia	Mild age-related changes.Minimal leukostasis in leptomeningeal vessels
**C048**	**68**	**M**	N/A	N/A	N/A	**1465**	**10**	Metastatic Colon Cancer	Mild age-related changes, microvascular pathology
**C049**	85	M	N/A	N/A	N/A	1189	23	Heart Failure, Myocardial Infarction	Mild ageing-related with AD-type changes (Braak Stage III)

ID	Age (y)	Sex	MS subtype	DD (y)	TOW (y)	BW (gr)	PMI (h)	Cause of Death	PM diagnosis
**PDC011**	71	M	N/A	N/A	N/A	1443	52	Hemopericardium, Ruptured Acute myocardial infarction	Mild hypoxic changes
**PDC013**	77	M	N/A	N/A	N/A	1628	48	Not Reported	Mild ageing-related changes
**PDC016**	91	F	N/A	N/A	N/A	1090	22	Bronchial Pneumonia, Old Age	Ageing-related changes. Calcification of the pallidus & anterior commissure
**PDC019**	74	F	N/A	N/A	N/A	1072	29	Not Reported	Mild ageing-related changes
**PDC022**	75	M	N/A	N/A	N/A	1489	12	Squamous Cell Carcinoma of Lung	Mild ageing-related changes
**PDC023**	78	F	N/A	N/A	N/A	1418	23	Not Reported	Mild ageing-related changes
**PDC026**	80	F	N/A	N/A	N/A	1269	23	Breast Carcinoma, Carcinosarcoma Uterus	Mild ageing-related with AD-type changes

Supplementary Table 1. Detailed clinical features of the MS and control cohort used in this study. (ID = identification ; M = male ; F = Female ; RRMS = relapse-remitting MS ; PPMS = primary progressive MS ; SPMS = secondary progressive MS ; MS = multiple sclerosis ; DD = disease duration ; TOW = time from onset to wheelchair ; BW = brain weight ; PMI = post-mortem interval : PM = post-mortem ; N/A = not applicable ; AD= Alzheimer’s disease)

Target	Reference	dilution	Clone	AR	Incubation
4G8 (Aβ, IF)	Biolegend SIG-39220	1:24000	Mouse Monoclonal	Formic acid 70%	ON RT
CD31 (IF)	Agilent M082301-2	1:25	Mouse Monoclonal	Microwave Citrate pH6	ON 4°C
CD34 (IF)	Serotec MCAP547	1:50	Mouse Monoclonal	Microwave Citrate pH6	ON 4°C
CD68	ProteinTech M0876	1:100	Mouse Monoclonal	Autoclave Citrate pH6	1h RT
GFAP	Dako Z0334	1:4000	Rabbit Polyclonal	Autoclave Citrate pH6	1h RT
GFAP (IF)	Abcam Ab4674	1:1000	Chicken Polyclonal	Autoclave Citrate pH6	1h RT
NeuN	Sigma-Aldrich MAB377	1 : 400	Mouse Monoclonal	Autoclave Citrate pH6	1h RT
NeuN	Abcam Ab177487	1:200 (IF)	Rabbit Monoclonal	Autoclave Citrate pH6	1h RT
NOR1	Thermo Fisher MA5-26703	1:100<br>1:100 (IF)	Mouse Monoclonal	Autoclave Tris-EDTA pH9	1h RT
Nurr1	Abcam Ab41917	1:1500	Mouse Monoclonal	Autoclave Tris-EDTA pH9	ON 4°C
PLP	Bio-Rad MCA839G	1:1000	Mouse Monoclonal	Microwave Citrate pH6	1h RT
p19^INK4d	Abcam ab262871	1:100	Rabbit Polyclonal	Microwave Citrate pH6	ON 4°C
p21^CIP1	Merck 05-655	1:3000	Mouse Monoclonal	Autoclave Citrate pH6	ON 4°C
TMEM119 (IF)	Sigma-Aldrich HPA051870	1:1500	Rabbit Polyclonal	Autoclave Tris-EDTA pH9	1h RT

Alexafluor Donkey anti-mouse 488nm	Thermo Fisher A-21202	1:100 (IF, secondary antibody)	Polyclonal	N/A	1h RT
Alexafluor Donkey-anti-rabbit 594nm	Thermo Fisher A-21207	1:100 (IF, secondary antibody)	Polyclonal	N/A	1h RT
Alexa Fluor Goat anti-Chicken 647nm	Life Technologies A21449	1:100 (IF, secondary antibody)	Polyclonal	N/A	1h RT

Supplementary Table 2. Antibodies used in staining procedures presented in this article. (RT = room temperature ; IF = immunofluorescence ; ON= overnight)
